# Fecal microbiome of pigs fed diets differing in protein and amino acid content raised in thermoneutral or cyclical heat stress conditions

**DOI:** 10.3389/fmicb.2025.1585374

**Published:** 2025-06-02

**Authors:** Marllon José Karpeggiane de Oliveira, Qinnan Yang, Antônio Diego Brandão Melo, Danilo Alves Marçal, Nate Korth, Natasha Pavlovikj, Andrew K. Benson, John Khun Kyaw Htoo, Henrique Gastmann Brand, Luciano Hauschild, Joao Carlos Gomes-Neto

**Affiliations:** ^1^Department of Animal Science, São Paulo State University (UNESP), School of Agricultural and Veterinary Sciences, Jaboticabal, São Paulo, Brazil; ^2^Department of Food Science and Technology, University of Nebraska-Lincoln, Lincoln, NE, United States; ^3^Nebraska Food for Health Center, University of Nebraska-Lincoln, Lincoln, NE, United States; ^4^Holland Computing Center, University of Nebraska-Lincoln, Lincoln, NE, United States; ^5^Evonik Operations GmbH, Hanau, Germany; ^6^Evonik Brasil Ltda., São Paulo, Brazil; ^7^Center for Food Animal Health, Department of Animal Sciences, The Ohio State University, Wooster, OH, United States

**Keywords:** 16S rRNA, gut microbiome, gut microbiota, high throughput sequencing, swine

## Abstract

The pig microbiome composition is affected by factors such as dietary changes, genetics, and diseases. Recent evidence suggests that housing temperature may also contribute to the variability in community structure and composition. Therefore, we investigated the interactive effects of different nutritional strategies and heat stress (HS) on the fecal microbiota composition, community structure, taxon distribution, and taxa correlation structure of pigs. Forty-eight (*Landrace* × *Large White*) finishing gilts with an average of 67.7 ± 6.2 kg of body weight (BW) were distributed in a 2 × 3 factorial arrangement: two temperatures [thermoneutral (TN, 22°C for 24 h) and cyclic heat stress (CHS, 12 h to 35°C and 12 h to 22°C)] and three diets varying in the dietary crude protein (CP) contents and amino acid (AA) levels [high CP (HP); low CP-free AA-supplemented diet (LPAA); low CP-free AA-supplemented diet and digestible Lys level (+20%), and Lys:AA ratios above recommendations (LPAA+)] originating six treatments (eight replicates of one pig). Pigs were fed *ad libitum* throughout the study. The 16S ribosomal RNA (rRNA)-based microbiome analysis was conducted in fecal samples collected on days 0 and 27 (endpoint). Overall, microbiome analysis suggested an increased richness in the fecal microbiome of pigs raised in TN conditions fed a diet supplemented with higher levels of AA (LPAA+). In addition, changes in the fecal microbiome composition indicated that *Mogibacterium* was significantly diminished in the feces of pigs fed the LPAA diet when compared to pigs fed the LPAA+, both in CHS conditions. *Oscillospira* was reduced in the feces of pigs fed a diet containing exclusively protein-bound as the source of AA, while the more the feed-grade AA was included in the remaining diets, the more the abundance of this taxon in fecal samples. Despite dietary alterations, C*orynebacterium* was enriched under CHS compared to TN, whereas the enrichment of *Prevotella* and *Eubacterium hallii* group was higher in the TN group. Outcomes of this study suggest that changes in fecal microbiota composition were mainly associated with temperature, pointing toward potential taxa that may contribute to physiological adaptation to heat stress.

## 1 Introduction

Modern swine commercial farms are populated with outbred genotypes that have a variable response to lean meat deposition and feed conversion, among which, heat stress (HS) can be an environmental factor capable of negatively altering performance and profitability (St-Pierre et al., 2003; Ross et al., 2015). Heat stress can affect all stages of swine production. Its net effect is primarily determined by the building characteristics, stocking density, and geography (climate influences), which are collectively responsible for creating the microclimate that influences air quality, barn temperature, and, consequently, pig growth and health (Godyń et al., 2020; Schauberger et al., 2020; De Prekel et al., 2024). Continuously exposing growing-finishing pigs to HS can negatively affect feed intake, lean meat deposition, and feed conversion variability, which are crucial economic metrics in modern swine production (Ross et al., 2015; de Oliveira et al., 2024). As such, it is known to a certain extent that HS reduces pig performance (de Oliveira et al., 2022, 2024), perhaps due to changes their digestive, absorptive, and post-absorptive metabolism of nutrients (Morales et al., 2016), and disruption of feeding behavior (de Oliveira et al., 2023). In addition, HS might reduce disease resistance by altering physiological immune responses, compromising the intestinal barrier (Gabler et al., 2018; Xiong et al., 2022), and ultimately predisposing pigs to intestinal inflammation often caused by endemic enteric pathogens that circulate in modern farms (Vidal et al., 2019; Luppi et al., 2023). Apart from its well-recognized detrimental results on production traits, HS has been reported to alter the gut microbiome composition and, consequently, influence colonization resistance against enteric pathogens and overall metabolism (Le Sciellour et al., 2019; Xiong et al., 2020; Gomes-Neto et al., 2023; Liao et al., 2024).

Dietary amino acid (AA) supplementation has been reported to be a potential attenuating factor to the deleterious effects caused by HS in pigs, especially regarding gut health and immune response. Jejunal damage due to HS was attenuated when finishing pigs were fed supplemental arginine (Yi et al., 2020), by partially helping pigs recover jejunum villi height (Morales et al., 2021). While arginine, histidine, and methionine contribute to restoring the intestinal epithelium integrity of the small intestine (Wu et al., 2009), threonine is used to produce a mucin glycoprotein (Faure et al., 2002) which helps to maintain intestinal mucosal integrity (Yi et al., 2017). Mucin-type O-glycans, the primary constituents of mucins, are known to be degraded by *Bacteroides thetaiotaomicron* and can affect mice's response in beneficial ways through mutualistic relationships as reported by Bergstrom and Xia (2013). Tryptophan is a precursor for synthesizing haptoglobin, an acute-phase protein secreted during an inflammatory process (Le Floc'h and Seve, 2007) and increases during HS (Santos et al., 2021). Likewise, diet varying in protein composition can also play a role in microbiome modulation, being responsible for direct changes in keystone taxa (e.g., *Blautia, Clostridium sensu stricto 1, Dorea, Lactobacillus, Mogibacterium, Peptococcus, Prevotella, Prevotellaceae NK3B31, Prevotellaceae UCG 001, Rikenellaceae RC9, Ruminococcus, Sarcina, Streptococcus, Terrisporobacter, Treponema*, and *Turicibacter*) present in pig fecal microbiome (Zhou et al., 2016; Sung et al., 2023). Modulation of gut microbiota at some specific genus, such as *Lactobacillus* (Kim et al., 2007), *Treponema* (Mäkinen and Mäkinen, 1996) has a prominent influence on AA metabolism because of its proteolytic capacity and AA utilization. Therefore, modifying the availability of protein levels as well as sources of AA (protein-bound and feed-grade) might be a useful tool to increase the availability of AA toward better utilization at the community level (Dai et al., 2013). Thus, the level of such micro-nutrients is expected to modulate the structure, composition, and functionality of the pig microbiota, to benefit HS-exposed pigs to enhance performance.

Previous studies have reported the effect of HS (Xiong et al., 2020; Hu et al., 2021, 2022; Xiong et al., 2022) and diet/feedstuff (Zhou et al., 2016; Liu et al., 2023) on the microbiome of pigs. However, limited information is available about their potential integrative effects in commercial growing pigs. Therefore, a more comprehensive understanding of how protein and AA levels may modulate the intestinal microbial gut ecosystem of pigs raised in HS conditions, compared to pigs raised in thermoneutrality (TN), is needed. Protein and AA appear to be useful tools to maximize pig performance and lean mass deposition, but the role of the gut microbiome in mediating gut health and host metabolism under stressful conditions remains largely unclear. Thus, we hypothesized that protein and AA content levels may modulate the fecal microbiota composition of pigs raised in cyclic heat stress (CHS) conditions differently from those in TN conditions. More specifically, we expected changes in some genus levels, such as *Clostridium, Prevotella, Fusobacterium, Lactobacillus, Treponema, Roseburia, Ruminococcus, Oscillospira, Blautia*, and *Megasphera*, known core members of the finishing pigs fecal microbiome (Holman et al., 2017; Wang et al., 2019, 2021; Wylensek et al., 2020; Arfken et al., 2020; Luo et al., 2022; Dong et al., 2023) which can influence community structure and function of the gut microbiota, being associated with worsening (e.g., *Clostridium* with species level largely unexplored) or enhancement (e.g., *Prevotella*) of pig performance. Therefore, our primary expectation was that there would be a direct modulation of putatively short-chain fatty acid (SCFA) producers, given that those molecules have the capacity to influence basal metabolism and gut health (Chi et al., 2024). To reach that goal, we performed 16S ribosomal RNA (rRNA) amplicon sequencing to map bacterial taxa that could differentiate between pigs, according to their nutritional strategies and/or housing temperature. In summary, our results suggest that under our experimental settings, changes in fecal microbiota composition were mainly associated with temperature changes, pointing toward potential taxa that may be needed for physiological adaptation to CHS or be reflective of a microbiome bottleneck.

## 2 Materials and methods

### 2.1 Animals, housing, management, and experimental design

All the experimental procedures and methods were reviewed and approved by the Institutional Animal Care and Use Committee at São Paulo State University (protocol No. 3380/20). This study used 48 selected crossbred (*Landrace* × *Large White*) finishing gilts (Agroceres PIC Camborough). Animals arrived at the Swine Research Facilities of São Paulo State University (Jaboticabal, São Paulo, Brazil) with an average of 24.5 ± 2.9 kg of body weight (BW). They were individually identified using ear tags and an exclusive electronic chip (plastic button tag containing passive radio-frequency identification transponders, which allows the animals access to electronic feeder stations). Pigs were randomly assigned to one of two similar rooms kept in TN housing conditions (22°C) and with a fixed artificial light photoperiod of 12 h (06:00–18:00 h) until they reached a BW of approximately 67 kg. Between arrival and the experiment beginning, regardless of the room, all pigs were fed *ad libitum* with a commercial diet formulated according to their nutritional requirements. They had free access to water, provided by low-pressure nipple drinkers. When pigs achieved an average of 67.7 ± 6.2 kg of BW, the house temperature of experimental room 1 was maintained at thermoneutrality (TN, 22°C, 24 h/day). In contrast, in experimental room 2, a CHS condition was set by fluctuating the ambient temperature from 22°C (20:01–07:59 h) to 35°C (08:00 to 20:00 h). Within each room, pigs were randomly assigned to one of the three diets varying in the dietary crude protein (CP) contents and AA levels, being (1) diet HP, high CP diet (17.19% CP), 0.78% of standardized ileal digestive (SID) Lys, AA:Lys ratios, Met+Cys 61.5%, Thr 70.5%, Trp, 23.1%, Val, 92.3%, and Ile 82.1%, without supplemental crystalline AA; (2) low CP-free AA-supplemented diet (LPAA), low CP diet (12.46% CP), supplemented with crystalline AA, SID Lys of 0.78%, AA:Lys ratios, Met+Cys 57.7%, Thr 62.8%, Trp 17.9%, Val 65.4 %, Ile 52.6%, and diet LPAA+, low CP diet supplemented with crystalline AA along with increased SID Lys level (+20%) and AA:Lys ratios (14.28% CP, SID Lys 0.94%, AA:Lys ratios, Met+Cys 65%, Thr 72%, Trp 20%, Val 70%, and Ile 54%). Therefore, a 2 × 3 factorial arrangement with two housing temperatures (TN and CHS) and three diets (HP, LPAA, LPAA+) resulted in six treatments. Each treatment had eight replicates of one pig each. The experiment lasted 27 days. A complete description of the animal study, such as body composition, nutrient intake, nitrogen balance, serum, and plasma metabolites concentration, from which the samples were taken, was previously described elsewhere (de Oliveira et al., 2022).

### 2.2 Sample collection and DNA extraction

At the beginning of the experiment (day 0) and at the end (day 27), before any other sample collection (i.e., rectal temperature), fecal samples for 16S rRNA-based microbiome analysis were collected. The fecal sample was chosen as a proxy for the large intestine gut microbiome composition and temporal sampling of live animals. Each day, a fecal sample was aseptically collected from the rectum of all pigs (48 pigs) after rectal stimulation. It was kept on ice during transport to the laboratory and stored at −80°C until further processing. Before DNA extraction, 250 mg of fecal sample was washed using 2 ml of sterile PBS (phosphate-buffered saline) and centrifuged once (4.5 min, 8 × 1,000 rcf). The supernatant was carefully discarded, and the formed pellet was used to repeat the procedure twice. The final pellet was used for DNA extraction following the manufacturer's instructions using the DNeasy PowerSoil Pro Kit (Qiagen, Hilden, Germany). All samples were eluted in 60 μL of an elution buffer and frozen at −80°C before quality assessment and sequencing. All samples were quality-checked for DNA concentration using a Nanodrop One spectrophotometer (Thermo Fisher Scientific, Inc., Middletown, VA, USA).

### 2.3 16S rRNA amplicon sequencing and bioinformatic processing

The V4 region of the bacterial 16S rRNA gene was amplified from each sample using Phusion High-Fidelity PCR Master Mix with HF Buffer (Thermo Scientific, Waltham, MA, USA), following the modified dual-indexing sequencing strategy (Kozich et al., 2013; Yang et al., 2022, 2023; Korth et al., 2024). Paired-end sequences were analyzed using the Quantitative Insights Into Microbial Ecology (QIIME2) program version 2, 2021.2 (Bolyen et al., 2019). Sequences were truncated (220 bases for forward reads and 160 bases for reverse reads) and denoised into amplicon sequence variants (ASVs) using deficiency of adenosine deaminase 2 (DADA2) (Callahan et al., 2016), then rarefied to 5,000 reads per sample, as previously described (Summers et al., 2019; Gonçalves et al., 2024). All ASVs were assigned taxonomic information using a pretrained sklearn-based taxonomy classifier SILVA reference database (*silva-138.1-ssu-nr99*) (Pedregosa et al., 2011; Quast et al., 2013). All sequencing can be found on NCBI (https://www.ncbi.nlm.nih.gov/bioproject/PRJNA985664, accessed on 20 June 2023). Before statistical analysis, only bacterial taxa (contained domain = “Bacteria”) containing genus-level information in the name (g from QIIME2 output) were filtered for both diversity and taxonomic analyses. For co-occurrence network analysis, information on the genus included in the *Clostridiaceae* family was collapsed together. A similar procedure was performed for the *Prevotellaceae* family. For the remaining analysis, information at the genus level was analyzed separately.

### 2.4 Network analysis for community structure

Co-occurrence networks were used to identify the central taxa of the treatment's community. Network construction and visualization were performed using the NetCoMi package (Peschel et al., 2021) within the R version 4.3.0 statistical framework. Overall, samples were normalized to 5,000 reads and grouped by day, temperature, and diet. According to the day of sample collection (0 and 27), each association-based network was constructed for each group or identified treatment by employing a centered log-ratio transformation and Spearman-based correlations between the 50 most abundant microbial taxa. Only the taxa pairs with an absolute association/dissimilarity >3 were used for sparsification. Data interpretation was summarized based on each node representing a bacterial taxon; the size of each node was scaled according to the eigenvector centralities of the community, and the different colors indicated the modules (clusters) calculated in network construction. The forest green and red edges represented the estimated positive and negative Spearman's correlations, respectively, whereas the edge thickness corresponded to the strength of the association. Edges representing a value < 0.5 were not shown. The three taxa with the highest degree of centrality were selected to highlight cluster-specific central taxa.

### 2.5 Statistical analysis

All bacterial taxonomic outputs from QIIME2 were processed for quality control, all the way to statistical modeling using R version 4.3.0. The tidyverse library (version 1.3.1) was used for data exploration, analysis, and visualization (exploratory analysis and final plotting). Only bacterial genus-level annotation was used in all statistical analyses. All analyses were conducted based on temperature (housing temperature) × diet (nutritional strategies) interactions (temperature slice diet and diet slice temperature). A one-way analysis of variance (ANOVA) was used to assess the effect of diets on each temperature, as well as the effect of temperature on each diet on alpha-diversity metrics (Shannon and Simpson's D indexes), beta-diversity decomposition analysis (PC1 or PC2), taxon distribution, and differential taxonomic biomarkers. All ANOVA models were performed using the aov() function, whereas all the *post hoc* comparisons were performed using the TukeyHSD() function. When the temperature × diet interaction was significant in the ANOVA model (*p* < 0.05), a Tukey test was used to perform *post hoc* comparisons of diets according to temperature.

Both Shannon and Simpson's D indexes of alpha-diversity were calculated with the diversity() function from the vegan library (version 2.6.2). Beta-diversity analysis was used to evaluate the dissimilarity among groups. It was calculated using the vegdist() function from the vegan library (version 2.6.2), while using Bray–Curtis's distance matrix and removing all missing values of the analysis. A permutational multivariate analysis of variance (PERMANOVA) was used to calculate the effect of day, diet, temperature, and their interactions on beta-diversity (Bray–Curtis distances), using the adonis2 (permutations = 999, method = “bray”) from the vegan library (version 2.6.2). A supplementary beta-diversity analysis was performed to evaluate the effect of noising taxa on the final results. Noising taxa were assumed to be those taxa that were not significant based on taxonomic biomarker relative abundance of the most abundant taxa, structure of core community, and linear discriminant analysis, effect size (analysis described below). Treatments were assessed for analysis of similarity (ANOSIM) based on Operational Taxonomic Units (OTUs) relative abundance and generated a test statistic R using the method = “bray” from the vegan library version 2.6.2. For the principal coordinate analysis (PCA), a classical multidimensional scaling model was used to reduce the data to three dimensions (three principal coordinates – PCs), using the cmdscale function (k = 3) from the stats library version 4.3.0. A 3D plot was performed using plot_ly packages (Sievert, 2020). Taxon distribution was evaluated by individual taxon-based relative abundance (proportion), calculated per pig while accounting for the day, temperature, and diet. Taxa with relative abundance >2% (cut-off) were considered the most dominant taxa in the community. The identification of differentiating taxa was evaluated by Linear Discriminant Analysis (LDA) Effect Size (LeFse) according to treatments and experimental days. The analysis was performed with the aid of the Galaxy platform with a Wilcoxon *p*-value adjustment to 0.05 and a threshold (cut-off) on the logarithmic LDA score for discriminative taxa set at 2. Taking into account all samples, a Δchange in taxa distribution over the experiment was calculated as follows:


$$\begin{array}{l} \Delta \text{change }\left(\%\right)\;=\;\frac{\begin{array}{c} \text{Relative abundance on day }27\\ -\text{ Relative abundance on day }0 \end{array}}{\text{Relative abundance on day }0} \end{array}$$

Based on the most abundant taxa, the structure of the core community, and differential taxa on LeFse analysis, a list of potentially differential taxonomic biomarkers was set. The Δchange among taxonomic biomarker relative abundance across groups was depicted as a heatmap using the transformed [log_2_ (f + 1)] relative abundance of those listed taxa. For taxa with zero mean counts for a given treatment, the value of 0.01 was added before the transformation was applied. Taxa from the list of potentially differential taxonomic biomarkers were individually tested using the ANOVA model, followed by *post hoc* comparisons, when necessary, as previously described.

Ultimately, Pearson's correlations between taxa were used to construct the heatmap using the corrplot and correlogram in R using the ellipse method while ordered by hierarchical clustering (Wei and Simko, 2024; Wright, 2022). Data interpretation was summarized based on each ellipsis having its eccentricity parametrically scaled to the correlation value. Ellipses sloping top to the right or top to the left indicate positive and negative correlations, for each trait pair, respectively. The shading of the ellipse also denoted the strength of the relationship. Correlations were significant if *p* < 0.05. The default parameters were used for calculations if not stated for all R functions.

### 2.6 Computational platforms

All 16S rRNA microbiome bioinformatic analyses were performed on Crane, one of the Linux high-performance computing clusters at the University of Nebraska Holland Computing Center (HCC) [https://hcc.unl.edu/].

## 3 Results

### 3.1 Experimental approach and analytical workflow

**Figure 1:**
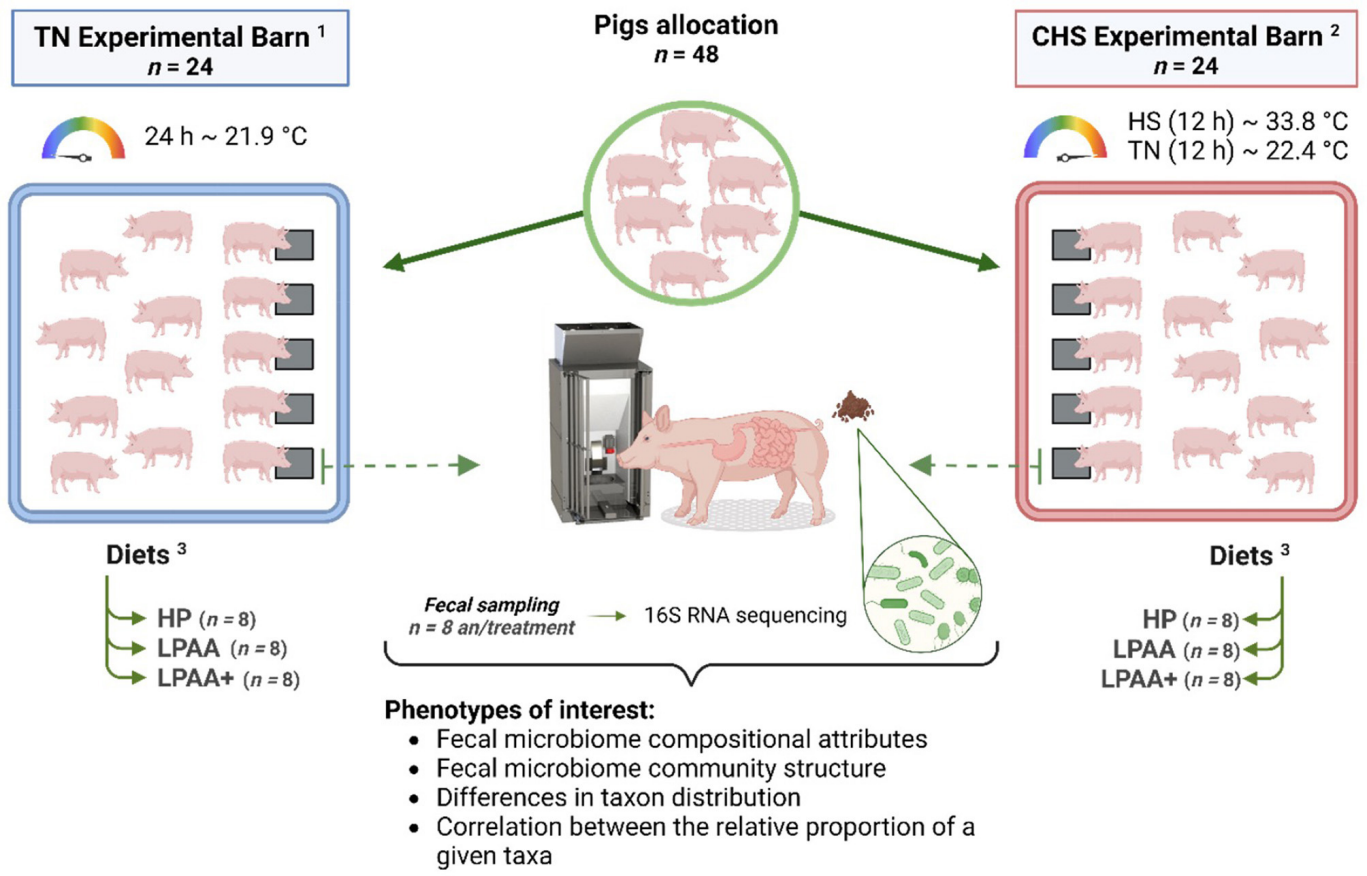
Cyclic heat stress study experimental design. Housing temperatures: ^1^TN, thermoneutrality (24 h, ~21.9°C) (*n* = 24) and ^2^CHS, Cyclic heat stress (12 h, ~33.8°C and 12 h, ~22.4°C) (*n* = 24). ^3^Diets: HP, high crude protein (CP) diet (*n* = 8); LPAA, low CP-free amino acid (AA) supplemented diet (*n* = 8); LPAA+ = low CP-free AA-supplemented diets and digestible Lys level (+20%), and Lys: AA ratios above recommendations (*n* = 8). Experimental design workflow including the number of animals per treatment, and phenotypes of interest: fecal microbiome composition and community structure including differences in taxon distribution, and correlation between the relative proportion of a given taxa.

**Figure 2:**
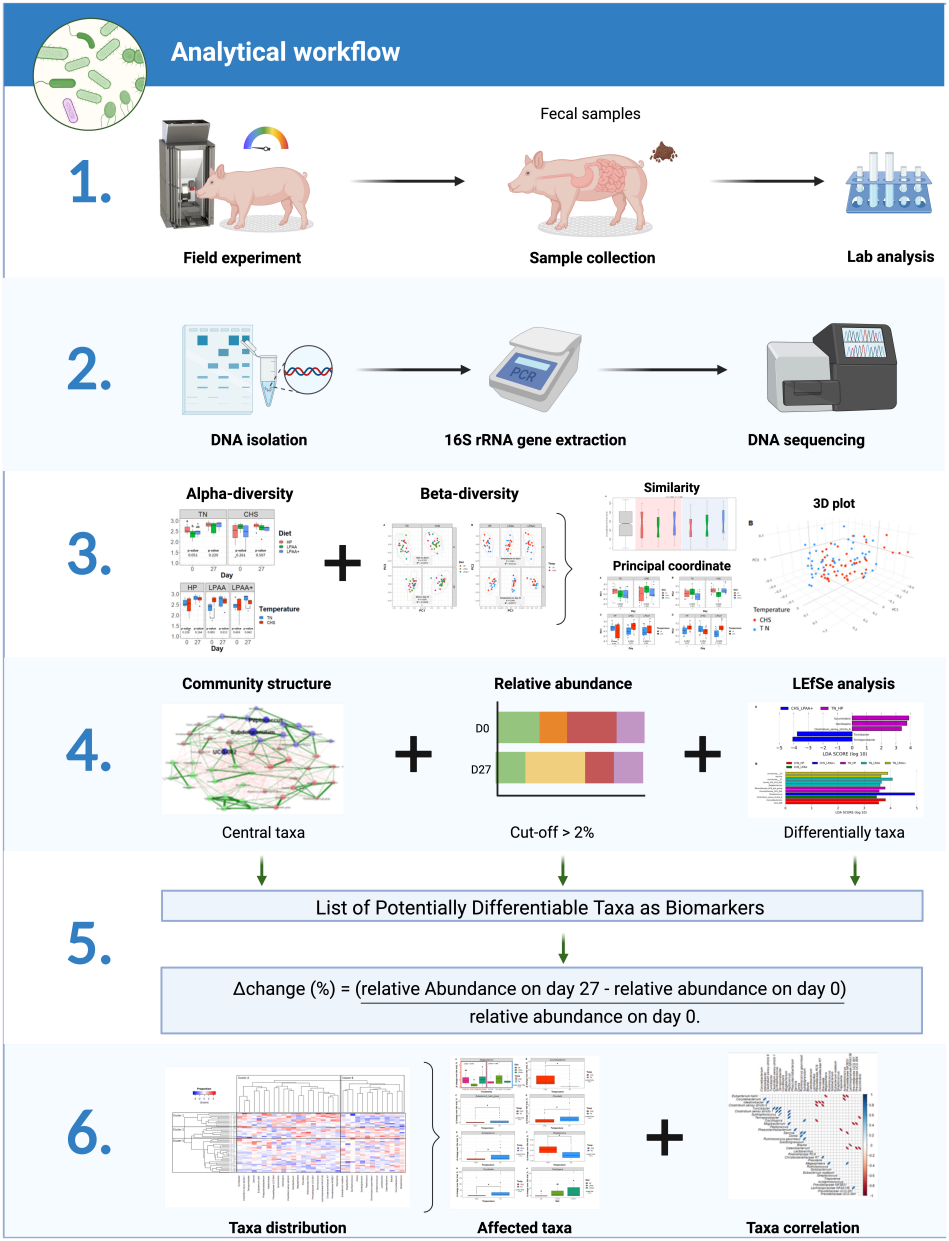
The analytical workflow included (1) community alpha-diversity (Shannon's and Simpson's D indexes) and beta-diversity (community composition, PERMANOVA for variance decomposition); based on (2) structure of core community, relative abundance and differentially abundant taxa (LeFse), a list of potentially differential taxonomic biomarkers was built and Δchange was calculated (3) and, (4) Taxa distribution (heat map), taxonomic differences (ANOVA), and taxa correlation (Pearson) were also evaluated.

In our previous assessment of these data (de Oliveira et al., 2022), we found that CHS negatively affected feed intake of pigs from days 0–27, and the AA supplementation was not capable of ameliorating this effect due to the absence of a diet × temperature interaction. Heat stress has also been reported to disturb the feeding behavior of pigs, modifying the size of the meal, the duration of each meal, and intervals between meals (de Oliveira et al., 2023). Given this, we conducted a 16S rRNA analysis based on microbiome analysis to evaluate the impact of Diet, Temperature, and their integrative effects on the fecal microbiome level. Figure 1 illustrates the experimental design and objectives of the study. In this randomized factorial design (2 × 3), the interaction between three different diets and two temperatures on fecal microbiome composition at an endpoint in relationship with the beginning of the study was evaluated. To our knowledge, this is the first study evaluating the fecal microbiome of pigs fed diets varying in protein and functional AA content and raised in CHS conditions—conditions that may be found in commercial conditions in swine production. To achieve our goals, we deployed the analytical workflow as shown in Figure 2, which systematically assessed community diversity, composition, and structure to identify major differentiating taxa across treatments.

### 3.2 Alpha-diversity

**Figure 3:**
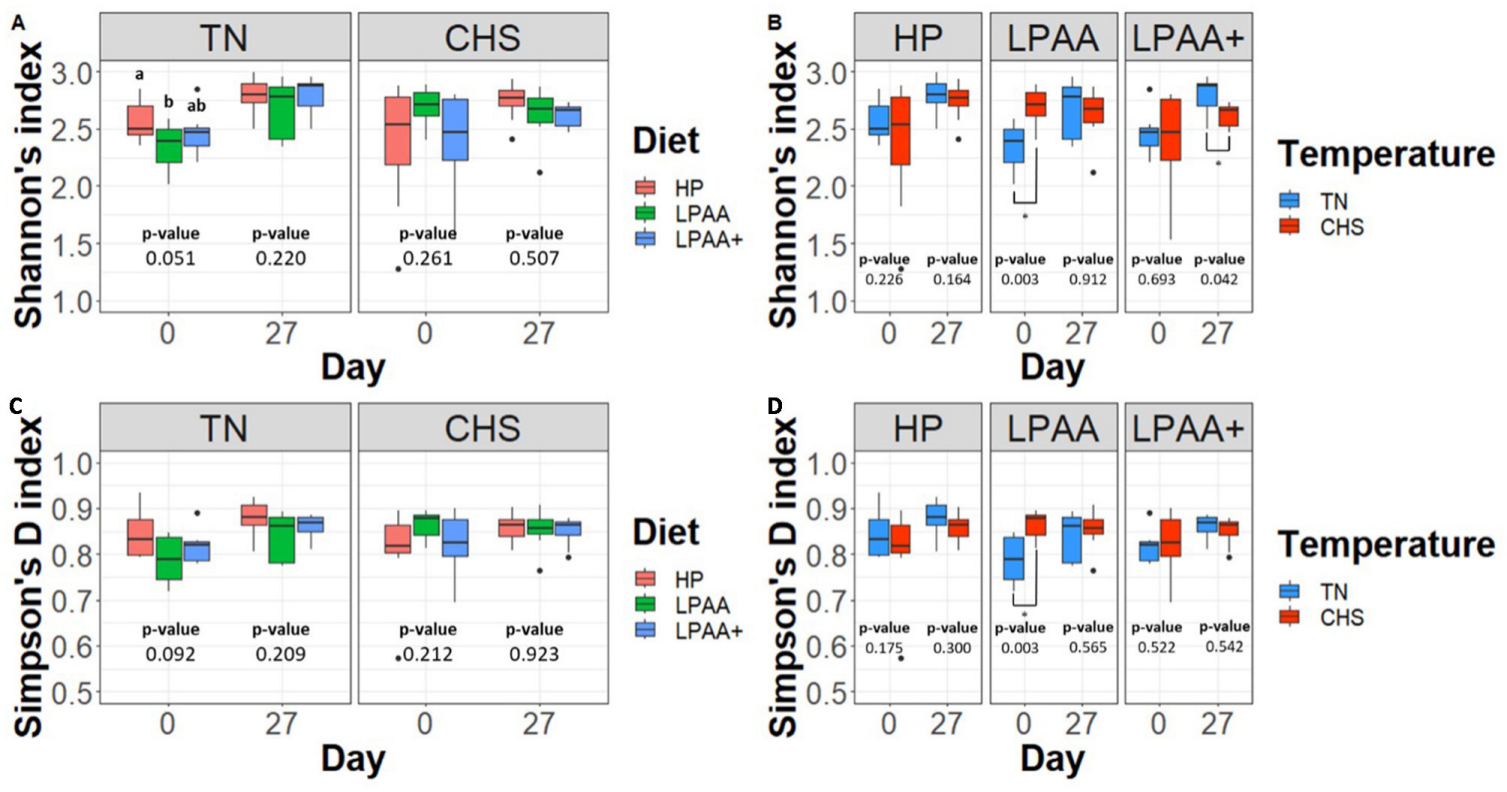
Alpha-diversity analysis (Shannon's and Simpson's D indexes) of the fecal microbiome composition of pigs. For both alpha-diversity metrics, a one-way ANOVA analysis was used to measure the effect of each studied factor (temperature, diet, and day) and their interactions. Housing temperatures: thermoneutrality and cyclic heat stress. Diets: HP, high crude protein (CP) diet; LPAA, low CP-free amino acid (AA) supplemented diet; LPAA+, low CP-free AA-supplemented diets and digestible Lys level (+20%), and Lys:AA ratios above recommendations. When the interaction temperature × diet was significant based on the one-way ANOVA analysis (*p* < 0.05), a Tukey test was used to perform *post hoc* comparisons for each temperature. Different superscript letters indicate significant differences between diets (*p* < 0.05). *Significant difference (*p* < 0.05) in housing temperature within diets. For both, Simpson's and Shannon's indexes, eight samples/treatment/day were used. **(A)** Shannon's and **(C)** Simpson's D indexes of alpha-diversity across pigs' diet at the beginning (day 0) and at the end (day 27) of the experiment, in each housing temperature, respectively. **(B)** Shannon's and **(D)** Simpson's D indexes of alpha-diversity across pigs' housing temperature, on days 0 and 27, in each diet, respectively. All treatments had equal fecal sample size (*n* = 8) on both days, except for TN LPAA+ on day 0 (*n* = 7). Each animal was considered an experimental unit throughout the analysis.

Alpha-diversity was measured using Shannon's (richness) and Simpson's (evenness) indexes of diversity, considering the factorial design and the percentual changes from days 0–27. As expected, regardless of temperature or diet effects, overall, on average, pigs increased the alpha-diversity fecal microbiome over time (day, *p* < 0.01, Supplementary Tables S1, S2). The significant diet × temperature interaction for Shannon's (*p* = 0.026, Supplementary Table S1) and Simpson's D (*p* = 0.022, Supplementary Table S2) index point out that, at the beginning of the trial, the baseline alpha-diversity differed among dietary groups in TN in terms of richness (Figure 3A, *p* = 0.051) and tend to differ in terms of evenness (Figure 3C, *p* = 0.092). The baseline alpha-diversity of pigs assigned to CHS did not differ. However, upon examining the Temperature effect for each dietary group, pigs of the LPAA group in CHS started the experiment with higher alpha-diversity (Figures 3B, D) than pigs raised in TN (*p* = 0.003). On day 27, pigs fed different dietary treatments raised in TN had similar richness (Figure 3A) and evenness (Figure 3C) to those raised in CHS. However, pigs fed LPAA+ and raised in TN had greater richness (*p* = 0.042) when compared to CHS pigs, with no difference in evenness (*p* = 0.542). It is worth highlighting that all the aforementioned initial differences in alpha-diversity of baselines no longer existed on day 27 (*p* > 0.05).

### 3.3 Beta-diversity

**Figure 4:**
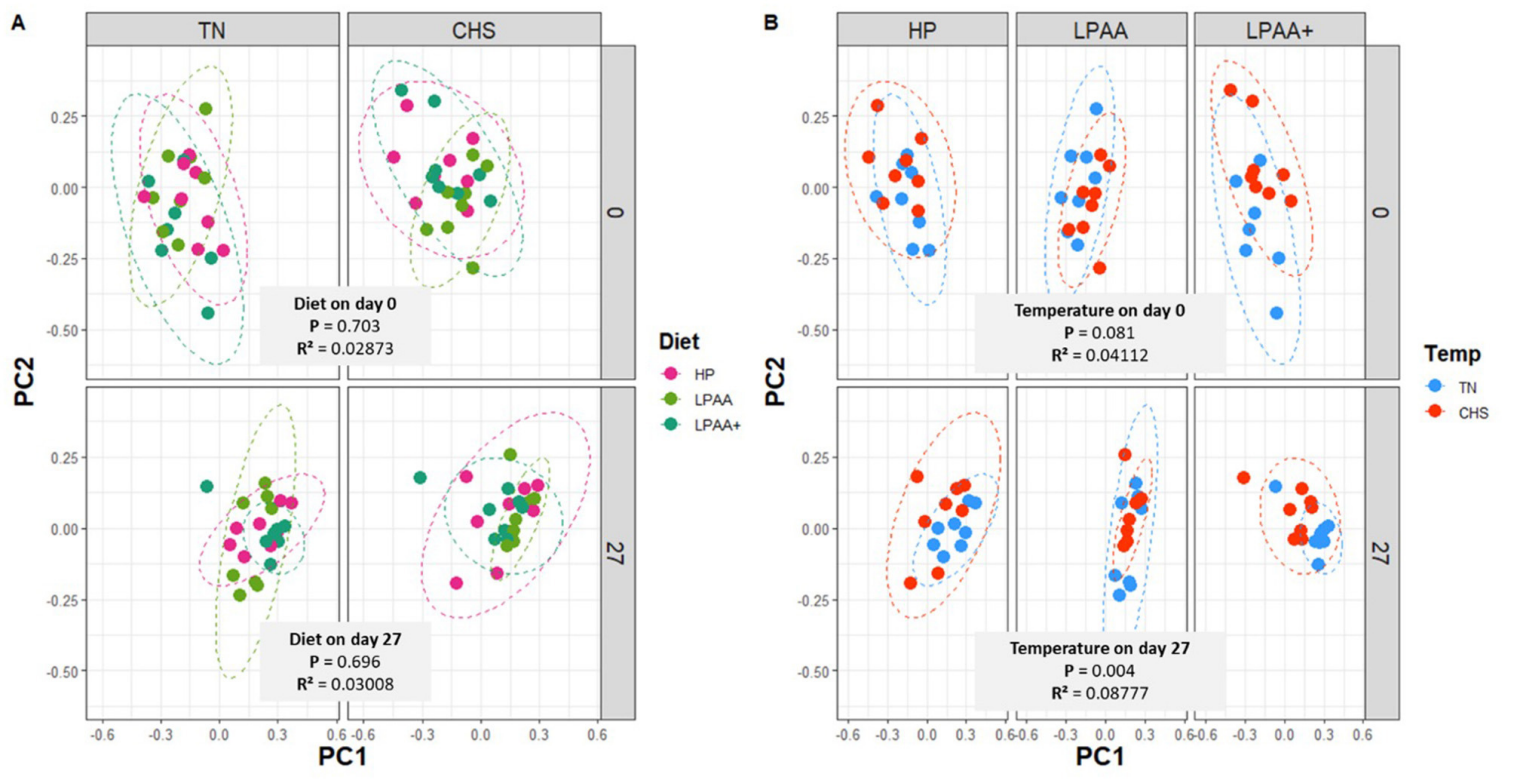
Beta-diversity analysis of the fecal microbiome composition of pigs. The Bray-Curtis distance matrix was used to calculate the beta-diversity between treatments. Housing temperatures: thermoneutrality and cyclic heat stress. Diets: HP, high crude protein (CP) diet; LPAA, low CP-free amino acid (AA) supplemented diet; LPAA+, low CP-free AA-supplemented diets and digestible Lys level (+20%), and Lys:AA ratios above recommendations. Two principal coordinates (PC1, PC2) are shown for fecal samples at the beginning (day 0) and the end (day 27) of the experiment, for each housing temperature **(A)**, and diet **(B)**. Testing all factors (and interactions) by PERMANOVA model supports diet × temperature interactions (*p* = 0.028, *R*^2^ = 0.03171), temperature (*p* = 0.006, *R*^2^ = 0.03002), and day (*p* = 0.001, *R*^2^ = 0.30642) effects, but not Diet (*p* = 0.487, *R*^2^ = 0.0127) effects (shown in Supplementary Table S3). Testing factors by the PERMANOVA model on Day 0 (shown in Supplementary Table S4) support diet × temperature interactions (*p* = 0.017, *R*^2^ = 0.09406), but not the single effect of diet (*p* = 0.703, *R*^2^ = 0.02873) or temperature (*p* = 0.081, *R*^2^ = 0.04112). Testing factors by the PERMANOVA model on day 27 (shown in Supplementary Table S5) support the temperature effect (*p* = 0.004, *R*^2^ = 0.08777) but not the diet effect (*p* = 0.696, *R*^2^ = 0.03008) or their interactions (*p* = 0.467, *R*^2^ = 0.03847). When the interaction diet × temperature was significant based on the PERMANOVA model (*p* < 0.05), a one-way ANOVA analysis was used to evaluate the beta-diversity decomposition analysis of diet and temperature (Supplementary Figure 1). All treatments had equal fecal sample size (*n* = 8), except for TN LPAA+ (*n* = 7). Each animal was considered an experimental unit throughout the analysis.

The beta-diversity PERMANOVA model (Supplementary Table S3) revealed a significant effect of temperature × diet (*p* = 0.028, *R*^2^ = 0.03171) and a tendency for temperature × day (*p* = 0.090, *R*^2^ = 0.01386) interaction. The main temperature (*p* = 0.006, *R*^2^ = 0.03002) and day (*p* = 0.001, *R*^2^ = 0.30642) effects were also captured by the analysis, supporting a major temporal change in the fecal microbiome composition (~30% explained by day alone). Upon splitting the analysis by day, the PERMANOVA model on day 0 (Supplementary Table S4) demonstrated a temperature × diet significant interaction (*p* = 0.017, *R*^2^ = 0.09406) and a tendency for temperature effect (*p* = 0.081, *R*^2^ = 0.04112), while on day 27, PERMANOVA model supported only the Temperature effect (*p* = 0.004, *R*^2^ = 0.08777). The PCA did not capture discrete differences in clusters on day 0 (*p* = 0.703, *R*^2^ = 0.02873) or even on day 27 (*p* = 0.696, *R*^2^ = 0.03008), reflecting overlapping compositional aspects. Beta-diversity composition, as analyzed by PC1 or PC2 separately, showed no significant changes in community dispersal (volatility), regardless of diet or day within each temperature (Supplementary Figures 1a, b, e, f). Although a trend in Temperature effects was observed for clusters at the baseline (*p* = 0.081, *R*^2^ = 0.04112), significant dissimilarity between TN and CHS groups was detected on day 27 (*p* = 0.004, *R*^2^ = 0.08777) (Figures 4A, B). The overall observed trend in temperature effects on day 0 was associated with more variability in community dispersal of pigs in LPAA+ diet group in CHS compared to TN pigs, as shown by the beta-diversity composition (PC2, *p* = 0.014, Supplementary Figure S1d). Nonetheless, on day 27, pigs fed LPAA+ diets raised under TN had a greater community dissimilarity than their counterparts raised in CHS (PC1, *p* = 0.045, Supplementary Figure S1g). A 3D plot of the three major axes generated by PCA, considering all samples, was depicted in Supplementary Figure S2 as a scatter plot, grouped by diet (Supplementary Figure S2a), temperature (Supplementary Figure S2b), and day (Supplementary Figure S2c), to support our analytical assessment.

**Figure 5:**
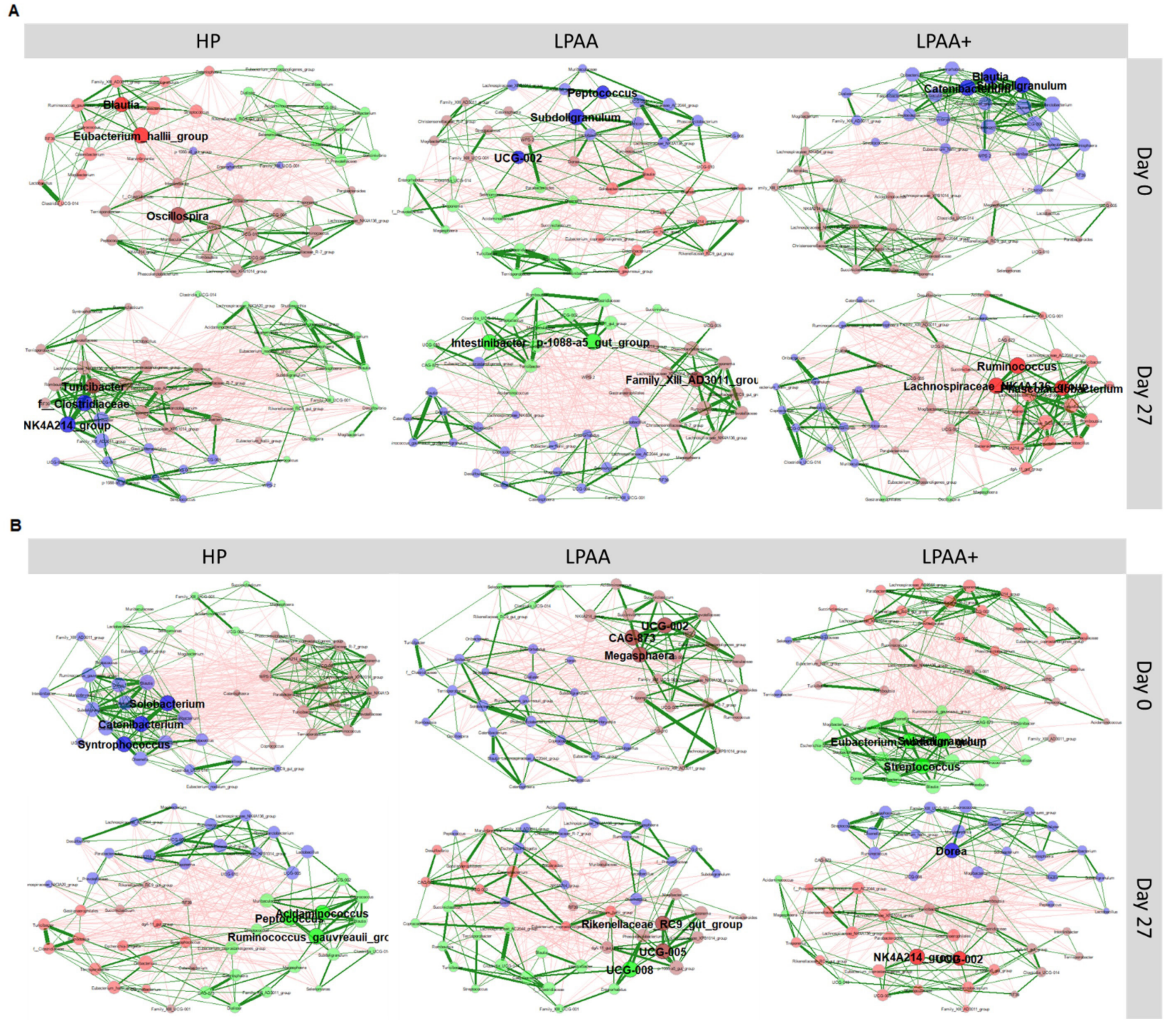
Structure of fecal microbiome community. The phylogenetic co-occurrence networks of pig's fecal microbiota at the beginning (day 0) and the end (day 27) of the experiment, according to dietary treatment for each housing temperature. Housing temperatures: **(A)** thermoneutrality and **(B)** cyclic heat stress. Diets: HP, high crude protein (CP) diet; LPAA, low CP-free amino acid (AA) supplemented diet; LPAA+, low CP-free AA-supplemented diets and digestible Lys level (+20%), and Lys:AA ratios above recommendations. Only the 50 most abundant microbial taxa were used to build the association-based networks. Associations between taxa were computed by Spearman's correlation. For sparsification, only taxa pairs with an absolute association/dissimilarity >3 were used. Node size is proportional to the eigenvector centralities of the community, and the different colors indicate the modules (clusters) within the network. The forest green and red edges color indicates the estimated positive and negative Spearman's correlations, respectively, while the edge thickness indicates the force of relationships. All treatments had equal fecal sample size (*n* = 8) on both days, except for TN LPAA+ on day 0 (*n* = 7). Each animal was considered an experimental unit throughout the analysis.

All the meaningful effects in terms of beta-diversity remained unaltered (Supplementary Tables S6–S8, Supplementary Figure S5), even after removing putatively “noising” taxa—categorized as taxa that were on average low in relative abundance (cut-off > 2%)—upon evaluating the core community structure (Figure 5), and by using a LeFse analysis to identify the more differentiable taxa (Supplementary Figure S4). In addition to PERMANOVA-based modeling, a non-parametric test (ANOSIM) was used (filtering or not the noising taxa—clarify that in parenthesis here) to determine whether differences between intertreatment groups were significantly greater than differences between intratreatment groups. The *R*-value of the unweighted unique fraction (UniFrac) rank on day 0 was *R* = 0.049, and *p* = 0.108 (Supplementary Figure S3a). The lack of significant statistical differences (*p* = 0.108) indicated no difference between the intertreatment groups. Therefore, no major dissimilarities in the microbial communities between treatments at the beginning of the experiment were observed. In contrast, the *R*-value of the unweighted UniFrac rank on day 27 was *R* = 0.107, and *p* = 0.008 (Supplementary Figure S3b), which indicated a difference in intertreatment groups, resulting in dissimilarity in the microbial communities between treatments at the end of the experiment.

### 3.4 Taxonomic changes

#### 3.4.1 Community structure and core-membership

Apart from the fecal community structure evaluated at the beta-diversity level, we used co-occurrence networks at the beginning and end of the experiment to identify the first three central taxa of the community of each treatment (Figure 5). At the same time, we assessed topology, interactions, and associations within the community. Taking together all treatments, at the beginning of the experiment (day 0), the unique central taxa were: *Blautia, Catenibacterium, Eubacterium hallii, Eubacterium nodatum, Megasphaera, Oscillospira, Peptococcus, Solobacterium, Streptococcus, Subdoligranulum*, and *Syntrophococcus*. At the end of the experiment (day 27), the unique central taxa observed across both days were *Acidaminococcus, Dorea, Intestinibacter, Lachnospiraceae NK4A136, Peptococcus, Phascolarctobacterium, Rikenellaceae RC9, Ruminococcus, Ruminococcus gauvreauii, Turicibacter*, and *Peptococcus*. A further evaluation of each treatment showed that, regardless of treatment, the central taxa at the end of the experiment (day 27) were different from those observed at the beginning of the experiment (day 0), with no hallmark of network alteration.

#### 3.4.2 Identifying major core taxa through relative abundances and enrichment analysis

On day 0, seven taxa (*Christensenellaceae R7, Clostridium sensu stricto 1, Lactobacillus, Megasphaera, Streptococcus, Terrisporobacter*, and *Treponema*) represented the most abundant taxa (cut-off > 2%), and accounted for around 74% of the community as a whole. On day 27, the most abundant taxa were *Christensenellaceae R7, Clostridium sensu stricto 1, Lactobacillus, Megasphaera, Prevotella, Prevotellaceae NK3B31, Prevotellaceae UCG-001, Streptococcus, Terrisporobacter, Treponema*, and *Turicibacter*, accounting for approximately 78% of the total community. It is worth highlighting that all the most abundant taxa on day 0 remained the most abundant taxa on day 27. Supplementary, according to LeFse results, demonstrated that *Clostridium sensu stricto 6, Oscillospira, Succinivibrio, Terrisporobacter*, and *Turicibacter* were discriminating between treatments on day 0 (Supplementary Figure S4a), whereas, on day 27, seven discriminative genera (*Clostridium sensu stricto 6, Corynebacterium, Mogibacterium, Prevotellaceae UCG 004, Rikenellaceae RC9, Sarcina*, and *Streptococcus*) were identified (Supplementary Figure S4b).

Collectively, our systematically analytical approach resulted in a list of 34 potentially differential fecal taxonomic biomarkers that could be differentially affected by diet and/or temperature over time, including *Acidaminococcus, Blautia, Catenibacterium, Christensenellaceae R7, Clostridium sensu stricto 1, Clostridium sensu stricto 6, Corynebacterium, Dorea, E. hallii, E. nodatum, Intestinibacter, Lachnospiraceae NK4A136, Lactobacillus, Megasphaera, Mogibacterium, Oscillospira, Peptococcus, Phascolarctobacterium, Prevotella, Prevotellaceae NK3B31, Prevotellaceae UCG 004, Prevotellaceae UCG 001, Rikenellaceae RC9, Ruminococcus, R. gauvreauii, Sarcina, Solobacterium, Streptococcus, Subdoligranulum, Succinivibrio, Syntrophococcus, Terrisporobacter, Treponema*, and *Turicibacter*.

### 3.5 Temporal changes across major taxa relative abundances

**Figure 6:**
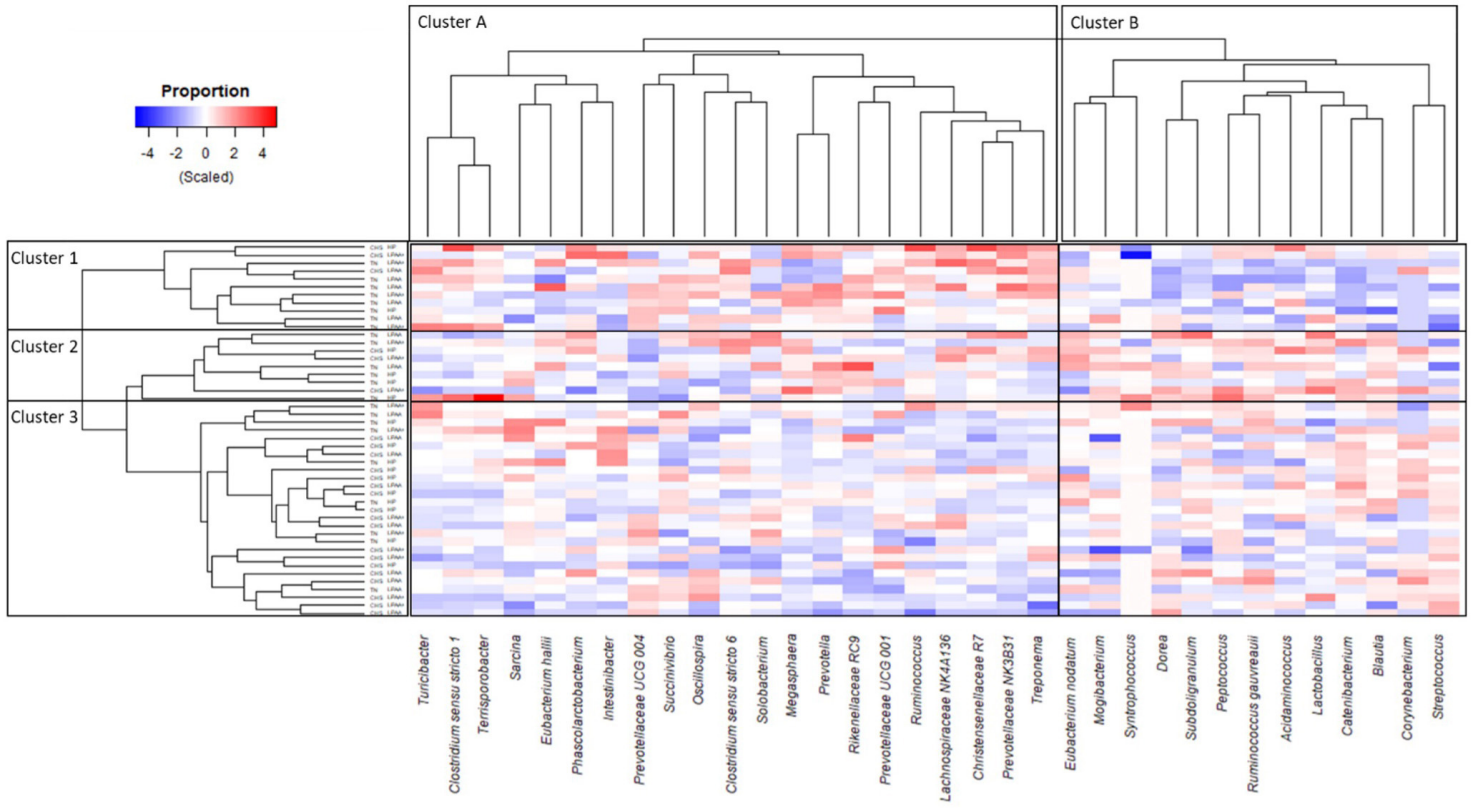
Heat map showing the differences in taxon distribution in pig's feces, based on Δchange of transformed [log_2_ (f + 1)] taxonomic biomarkers of the most abundant taxa (cut-off > 2%), the structure of core community (Figure 5), and Linear Discriminant Analysis (LDA), Effect Size (LeFse) (Supplementary Figure 4). The Δchange was calculated as follows: Δchange (%) = (relative abundance on day 27—relative abundance on day 0)/relative abundance on day 0. Housing temperatures: thermoneutrality (TN) and cyclic heat stress (CHS). Diets were HP, high crude protein (CP) diet; LPAA, low CP-free amino acid (AA) supplemented diet; LPAA+, low CP-free AA-supplemented diets and digestible Lys level (+20%), and Lys:AA ratios above recommendations. All treatments had equal fecal sample size (*n* = 8), except for TN LPAA+ (*n* = 7). Each animal was considered an experimental unit throughout the analysis.

The heat map (Figure 6) depicts the taxa distribution over time (Δchange) and shows a partial separation of TN and CHS pigs at the individual animal level. Overall, the hierarchical clustering divided the pigs into three main clusters: (1) 8 TN pigs (72.7%) and 3 CHS pigs (27.3%) with a clear separation, (2) 6 TN pigs (66.7%) and 3 CHS pigs (33.3%) clustering together but less distant from the next cluster, and (3) encompassing 9 TN pigs (33.3%) and the remaining 18 CHS pigs (66.7%). The taxa group, which comprised *Acidaminococcus, Blautia, Catenibacterium, Corynebacterium, Dorea, E. nodatum, Lactobacillus, Mogibacterium, Peptococcus, R. gauvreauii, Streptococcus, Subdoligranulum*, and *Syntrophococcus*, was found to be most abundant in the first cluster (Figure 6, Cluster 1b). In contrast, the taxa group comprised of *Christensenellaceae R7, Clostridium sensu stricto 1, Clostridium sensu stricto 6, E. hallii, Intestinibacter, Lachnospiraceae NK4A136, Megasphaera, Oscillospira, Phascolarctobacterium, Prevotella, Prevotellaceae NK3B31, Prevotellaceae UCG 004, Prevotellaceae UCG 001, Rikenellaceae RC9, Ruminococcus, Sarcina, Solobacterium, Succinivibrio, Terrisporobacter, Treponema*, and *Turicibacter* were found to be most abundant in the third cluster (Figure 6, Cluster 3a). These findings revealed the microbiota compositional changes in response to temperature, since cluster 1 was mainly composed of TN pigs, whereas cluster 3 was composed of CHS pigs.

### 3.6 Statistical assessment of core biomarker taxa and their correlation structure

**Figure 7:**
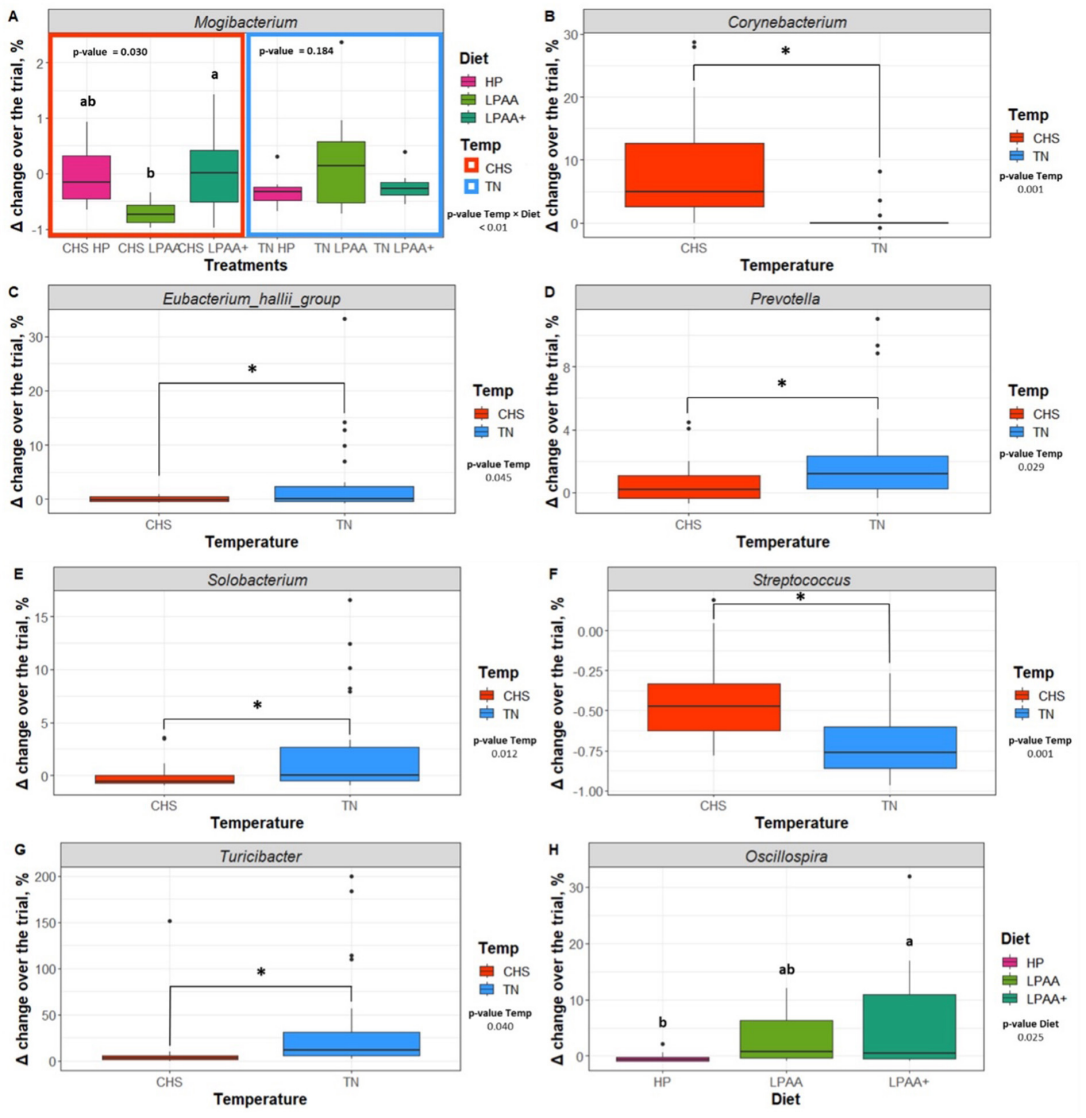
Differential taxonomic biomarkers (**A** - *Mogibacterium*, **B** - *Corynebacterium*, **C** - *Eubacterium hallii group*, **D** - *Prevotella*, **E** - *Solobacterium*, **F** - *Streptococcus*, **G** - *Turicibacter*, **H** - *Oscillospira*). Delta (Δ) change [log_2_ (f + 1)] of the proportion of taxonomic biomarkers of pig's fecal microbiome affected by temperature (Temp), Diet, or their interaction over the experiment. Effects were evaluated in selected significant taxa based on Δ change taxonomic biomarker relative abundance of the most abundant taxa (cut-off > 2%), the structure of core community (Figure 5), and Linear Discriminant Analysis (LDA), Effect Size (LeFse) (Supplementary Figure 4). Tests for all non-significant selected taxa are shown in Supplementary Table S9. The Δ change was calculated as follows: Δ change (%) = (relative abundance on day 27—relative abundance on day 0)/relative abundance on day 0. Housing temperatures: thermoneutrality (TN) and cyclic heat stress (CHS). Diets: HP, high crude protein (CP) diet; LPAA, low CP-free amino acid (AA) supplemented diet; LPAA+, low CP-free AA-supplemented diets and digestible Lys level (+20%), and Lys:AA ratios above recommendations. Statistical analysis was performed using the ANOVA model. When the temperature × diet interaction was significant (*p* < 0.05), a Tukey test was used to perform *post hoc* comparisons. Different superscript letters indicate significant differences between diets according to housing temperature.

**Figure 8:**
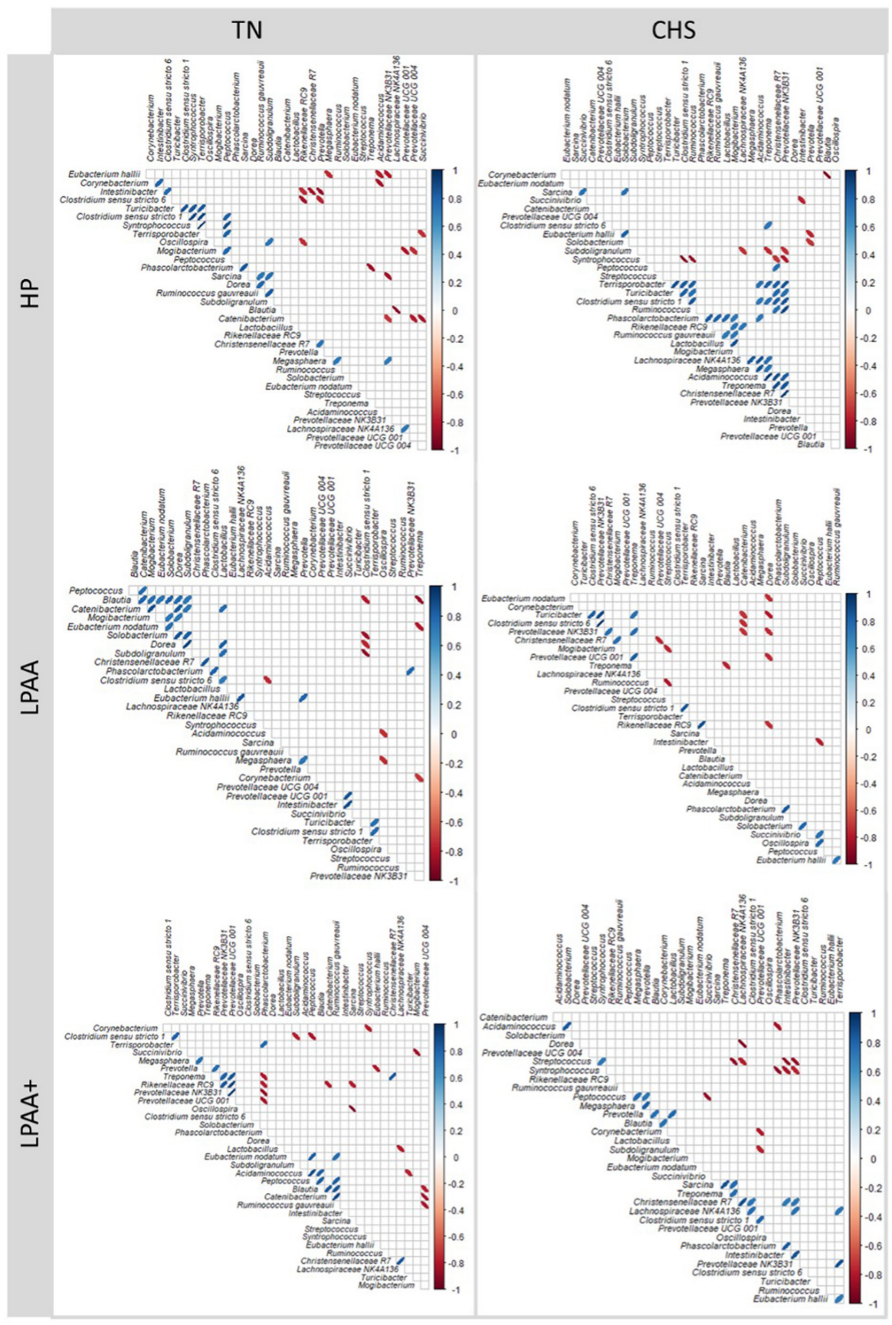
Correlation structure analysis [log_2_ (f + 1)] of selected significant taxa in pig's feces based on Δchange taxonomic biomarker relative abundance of the most abundant taxa (cut-off > 2%), the structure of core community (Figure 5), and Linear Discriminant Analysis (LDA), Effect Size (LeFse) (Supplementary Figure S4). The Δchange was calculated as follows: Δchange (%) = (relative abundance on day 27—relative abundance on day 0)/relative abundance on day 0. Pearson's correlation coefficients are expressed by an ellipse, reordered by hierarchical clustering for each temperature and each diet. Temperatures: thermoneutrality (TN) and cyclic heat stress (CHS). Diets were HP, high crude protein (CP) diet; LPAA, low CP-free amino acid (AA) supplemented diet; LPAA+, low CP-free AA-supplemented diets and digestible Lys level (+20%), and Lys:AA ratios above recommendations. The ellipses have their eccentricity parametrically scaled to the correlation value. Ellipses sloping top to the right or top to the left indicate positive and negative correlations (*p* < 0.05), for each trait pair, respectively. The shading of the ellipse also denotes the strength of the relationship. All treatments had equal fecal sample size (*n* = 8), except for TN LPAA+ (*n* = 7). Each animal was considered an experimental unit throughout the analysis.

Further assessment of differentiable taxa across treatments was performed using a one-way ANOVA model (Supplementary Table S9), and the most important taxa were depicted in Figure 7. *Mogibacterium* was identified as significantly differentiable by diet × temperature interaction (Figure 7A, *p* < 0.01). In the CHS condition, the feces of pigs fed the LPAA+ diet had a higher Δchange of *Mogibacterium* than that of pigs fed the LPAA diet, which showed a negative Δchange (reduction in proportion) over time. Feces of pigs fed the HP diet had an intermediate Δchange (*p* = 0.030). Under TN conditions, no diet effect was observed for the *Mogibacterium* (*p* = 0.184). In terms of Temperature effects and considering the TN group as a control (non-limiting condition of housing temperature), the feces of pigs raised under CHS conditions increased the relative abundance of *Corynebacterium* (Figure 7B), when compared to the feces of TN pigs (*p* < 0.001). An absence of change in the relative abundance of the *E. hallii group* under the CHS condition (Figure 7C) and a slight increase under TN conditions was detected (*p* = 0.045). For *Prevotella* (Figure 7D, *p* = 0.029) and *Turicibacter* (Figure 7G, *p* = 0.040), a slight change in the relative abundance for the CHS condition was observed when compared to the changes observed in pigs raised under TN conditions. *Solobacterium* (Figure 7E) reduced the relative abundance in the CHS condition compared to TN (*p* = 0.012). *Streptococcus* (Figure 7F) was decreased in the feces of TN pigs when compared to the feces of CHS pigs (*p* < 0.001). As for the diet effect, pigs fed the LPAA + diet (Figure 7H) increased the relative abundance of *Oscillospira* compared to pigs fed the HP diet, which reduced the proportion of this taxon. The feces of pigs fed the LPAA diet had an intermediate change in relative abundance (*p* = 0.025) for *Oscillospira*. In this study, temperature had the most substantial statistical effect across individual taxa, driving community structure changes over time. Significant positive and negative correlations were found between taxa, which were considered the major core taxa present across treatments over time and potentially identified as differential taxonomic biomarkers. Overall, regardless of treatment, the number of stronger positive Pearson's correlation values was more pronounced than negative Pearson's correlation values (Figure 8).

## 4 Discussion

Despite the emerging concern about HS in livestock, effective changes in modern swine production operations require dramatic changes in infrastructure, which can be cost-prohibitive at first or ever. However, alternative strategies related to nutrition in terms of dietary level of nutrients (e.g., reduced crude protein and increasing in functional AA), have been studied aiming at maximizing performance metrics (feed intake, feed conversion, and meat deposition) for herds raised under stressful situations (Morales et al., 2018; de Oliveira et al., 2022). Taking into account (1) the potential modulatory effect of protein and functional AA levels on the intestinal microbial ecosystem (Lin et al., 2017), highlighting that diet is an essential factor in controlling the composition and metabolic activities of the microbiota, and (2) the limited information regarding the interactive effects between the host, diet and temperature (HS); we sought to evaluate the fecal microbiome composition, community structure, taxonomic distribution, and taxa correlation across pigs housed under different temperatures fed diets enriched with functional amino acids expected to help pigs overcome HS situations. A primary goal of this study was to identify a set of fecal microbiome taxa under the influence of diet or temperature effects, aiming to understand which bacteria could be responsible for physiological adaptation to HS. Based on a robust and systematic analytical pipeline, and under our experimental conditions, we identified a list containing 34 potential biomarkers (possibly even functionally keystone taxa), among which known members of the swine core fecal microbiome for growing pigs were prominently present: *Acidaminococcus, Blautia*, C*orynebacterium, Clostridium, Dorea, E. hallii, Intestinibacter, Lactobacillus, Megasphaera, Oscillospira, Prevotella, Ruminococcus, Solobacterium, Streptococcus, Subdoligranulum, Succinivibrio, Treponema*, and *Turicibacter* (Holman et al., 2017; Wang et al., 2019, 2021; Li et al., 2020; Luo et al., 2022; Dong et al., 2023).

Among all major differentiable fecal microbiome taxa found in this study, *E. hallii group, Prevotella, Solobacterium*, and *Turicibacter* taxa were pronouncedly affected by Temperature, being enriched over time under the thermal neutral condition. *Prevotella* is one of the most predominant genera across the large intestine of pigs (Looft et al., 2014; Holman et al., 2017) and is a keystone taxon (increased abundance post-weaning) as it has a profound influence on the community structure and function of the gut microbiota in pigs. *Eubacterium* has also been reported as a predominant genus in the microbiota of pigs (Xiao et al., 2016). Whereas *Prevotella* is capable of producing succinate and acetate (Franke and Deppenmeier, 2018; Amat et al., 2020; Iljazovic et al., 2020) as an end product of anaerobic microbial fermentation, and *Eubacterium* is capable of producing butyrate (Levine et al., 2013), although both genera are associated with the production of propionate (Strobel, 1992; Engels et al., 2016; Trachsel et al., 2022; Sebastià et al., 2024). Propionate may strengthen intestinal barrier, promote immune cell function, energy source for the host apart from colonocytes, which typically use butyrate, and may affect the secretion of satiety hormones that can affect feed consumption (Zhang et al., 2019, 2022; Zhao et al., 2019; Verbeek et al., 2021; Yang and Zhao, 2021; Vasquez et al., 2022; Andrani et al., 2023; Pandey et al., 2023; Rathert-Williams et al., 2023). Producing succinate by *Prevotella* may also promote glucose homeostasis through intestinal gluconeogenesis (De Vadder et al., 2016). However, we lack further understanding of both species and strain-level diversity of these microbes, which may refine our understanding of community assembly and responsiveness to dietary changes, as research continues to make progress in this area. For instance, at least two species of *P. copri* and *P. stercorea* are expected to be present in the pig gut microbiome (Amat et al., 2020), as in humans (Yeoh et al., 2022). Phylogenetic analysis of *P. copri* genomes from human isolates suggests distinguishable geographical signatures that might be due to dietary changes in the Western population (Tett et al., 2019), which, combined with *in vitro* studies, points toward variability in fiber structural utilization across strains (Fehlner-Peach et al., 2019)—the central ecological premise for a novel symbiotic developmental strategy. *Prevotella* is also negatively associated with other quantitative traits, such as shedding of *Salmonella* Typhimurium and the Monophasic variant of *S*. Typhimurium in pigs, which could affect performance through inflammation, and food safety by contamination of products postslaughter, pointing to a potential pleiotropic beneficial effect of this keystone member of the swine microbiome post-weaning (Bearson et al., 2013; Naberhaus et al., 2020; Gomes-Neto et al., 2023; Kempf et al., 2023). Regarding the link between animal production traits and microbiota, *Prevotella* has shown a positive association with feed intake (Yang et al., 2018) and body weight gain (Mach et al., 2015), while *Eubacterium* has demonstrated a strong positive correlation with body weight, body weight gain, and fecal content of butyrate (Oh et al., 2020; Xu et al., 2021), It suggests that both genera may have a role in mediating pigs growth performance. In practice, that translates to a necessity to consider the ecology and genetic diversity of a given species to develop host-adapted symbiotic strategies, considering also the scope of the phenotype in question (e.g., colonization resistance, host metabolism, etc.) (Walter et al., 2018; Hitch et al., 2022).

In our experimental model, CHS also evoked an enrichment of *Corynebacterium* over time. It should be noted that this enrichment was the most expressive among all the major differentiable fecal microbiome taxa enriched for heat-stressed pigs. *Corynebacteria* are typically present in the genital tract of pigs (Poor et al., 2017), and the upper respiratory tract and are often non-pathogenic (Yibin et al., 2016). Considering that HS may trigger mucosal damage and decline disease resistance by compromised immune responses (Gabler et al., 2018; Xiong et al., 2022), which can predispose pigs to opportunistic pathogens such as *Corynebacteria* (Bernard, 2012; Tauch et al., 2016). Therefore, a deeper understanding of the role of this genus for pigs under HS conditions is encouraged, especially because pigs might be asymptomatic carriers of an array of *Corynebacterium* species (Poor et al., 2017), for example, the potentially zoonotic species *C. ulcerans, C. confusum*, and *C. amycolatum* (Schuhegger et al., 2009; Boschert et al., 2014). The emerging data on multidrug resistance in this genus (Poor et al., 2017) cause concern for animal and public health.

*Turicibacter* appears to be a biomarker for autism spectrum disorder (Gerges et al., 2024), therefore being associated with gut-brain axis function (Borsom et al., 2023), since it is predicted to impact intestinal serotonin secretion and neurotransmission (Wang et al., 2020; Lin et al., 2023). Serotonin requires tryptophan for its synthesis, and signaling is regulated by G-protein-coupled receptors (GPCRs) and ligand-gated cation channel heteropentameric receptors, which can influence gut motility as well (Gershon and Tack, 2007; Masson et al., 2012; Shah et al., 2021). As with *Prevotella*, the genetic diversity of *Turicibacter* strains may differentially affect their gut role by altering bile acid production, serum cholesterol, triglycerides, and adipose tissue mass in mouse models (Lynch et al., 2023). In a study of social stress in pigs, Nguyen et al. (2023) found *Turicibacter* to be enriched in pigs under stress, suggesting potential participation in physiological adaptation, which could be the case in our study since pigs under HS were not enriched with it.

In agreement with the outcomes of the present research, Hu et al. (2021) reported that *Streptococcus* and *Clostridium* were the top two taxa in the total distribution of the colon microbiota of HS pigs. Although Xiong et al. (2022) described an increase of *Streptococcus* in the microbiome of HS pigs, the present study reported a slight reduction over time of *Streptococcus* in pigs under cyclic HS, when compared to the reduction observed for pigs raised under thermoneutrality. *Streptococcus* has been reported to cause intestinal inflammation and apoptosis in pigs raised in HS conditions (Xiong et al., 2022), which leads to impaired performance. However, further studies on species and genetic diversity are needed.

With the advancement of technology-based omics, recent research has accumulated evidence demonstrating the influence of the gut microbiota on feeding behavior and energy metabolism through the communication axis between the gut and liver (Ringseis et al., 2020). Several gut-derived compounds may play a role in the gut-microbiota host communication, such as bile acids, microbial-associated molecular patterns, methylamines, amino acid-derived metabolites, and SCFA (Ringseis et al., 2020; Ringseis and Eder, 2022). Therefore, any microbiome disturbance might trigger changes in the ratio of commensal/mutualistic intestinal bacteria and substantially influence the production of these compounds, upregulating or downregulating the peripheral feedback signals' secretion. For instance, SCFAs are reported to stimulate the peripheral secretion of leptin, an acute-appetite-inducing signal, which is tightly regulated according to energy homeostasis and regulates several physiological processes, such as feeding behavior and metabolic rate (Xiong et al., 2004). Also, SCFA affects the secretion of incretins, such as peptide YY and GLP-1 and glucagon-like peptide 1 (Lin et al., 2012), which are acute satiety-inducing signals secreted during the preabsorptive phase upon sensing of feed or specific nutrients like amino acids (Ringseis et al., 2020). Therefore, the contradictory effects of SCFA on feed intake regulation cannot be associated solely with the increase or decrease in production by itself. Instead, the compositional profile of SCFA matters because the extent to which each specific SCFA contributes to feed intake regulation is different (Lin et al., 2012). It might likely help to justify the drop in feed intake reported for the heat-stressed pigs from where the samples for this study were obtained (de Oliveira et al., 2022), supported by other trials that linked a reduction in feed intake with changes in gut microbiota, and hence the profile of SCFA produced from microbial fermentation (Xia et al., 2022; Xiong et al., 2020; Hu et al., 2021).

Studies have explored the role of gut microbiota in pig feeding behavior (He et al., 2022a) and shown the sensitivity of microbiota to changes in ambient temperature (He et al., 2019; Xiong et al., 2020; Le Sciellour et al., 2019). However, the in-depth literature analysis on the interaction between microbiome, feeding behavior, and heat stress. Although the feeding behavior in conjunction with gut microbiome has been investigated to be used as a predictor of body composition traits of finishing pigs (He et al., 2022b), the field is just beginning to explore the role of gut microbiome interacting with feed behavior in heat stress conditions and the role of the gut-brain axis on it.

Our unique experimental approach with the ability to predictably alter environmental temperature and measure individual animal feed intake, provides some future great opportunities that are worth highlighting: (1) the need to increase the sample size and more evenly spaced sampling of the fecal microbiome for correlative analysis with feed intake and behavioral data; (2) the need to consider how randomization is performed at the beginning of a study since it can create a bias due to the initial microbiome composition (might become a blocking factor, for example, testing HS in two groups: high in *Prevotella* + *Turicibacter* vs. low in *Prevotella* + *Turicibacter*) to predictably access how dietary changes and housing under (high vs. normal vs. low) temperature would influence changes of such taxa over time; (3) the need for qPCR for absolute quantification of target bacteria and more detailed temporal dynamics (the use of qPCR also allows for increasing the sample size); (4) the need for isolation of such taxa and whole-genome sequencing for further characterization and creating a collection of potential probiotics; and (5) the need to get more species level information for community and taxa level analysis potentially using deep metagenomics in selected samples and do functional measuring through transcriptomics.

## 5 Conclusion

This uniquely designed study identified a collective list of bacterial genus taxa that may individually vary in the predictive power but collectively assist in discerning between animals undergoing CHS and those that are not. Among the strongest predictors are *Prevotella, E. hallii, and Turicibacter*, which might even be associated with physiological adaptation to CHS or reflect a microbiome bottleneck that could be causatively addressed through future studies controlling for initial microbiome composition. This suggests a potential role for SFCA and serotonin in regulating gut function during CHS, since these are expected metabolic products of those taxa. Therefore, we propose that our experimental approach and microbial quantification pipeline can be refined and expanded to achieve such goals.

## Data Availability

The original contributions presented in the study are publicly available. This data can be found here: https://www.ncbi.nlm.nih.gov/, accession number: PRJNA944612.
