# Supplementary material for: Fecal microbiome of pigs fed diets differing in protein and amino acid content raised in thermoneutral or cyclical heat stress conditions
Source: Front Microbiol. 2025 Jun 2;16:1585374. doi: 10.3389/fmicb.2025.1585374 (PMC12169254; doi:10.3389/fmicb.2025.1585374)
Supplement: Supplementary file 1 [file Data_Sheet_1.docx]

**SUPPLEMENTARY FILES**

**
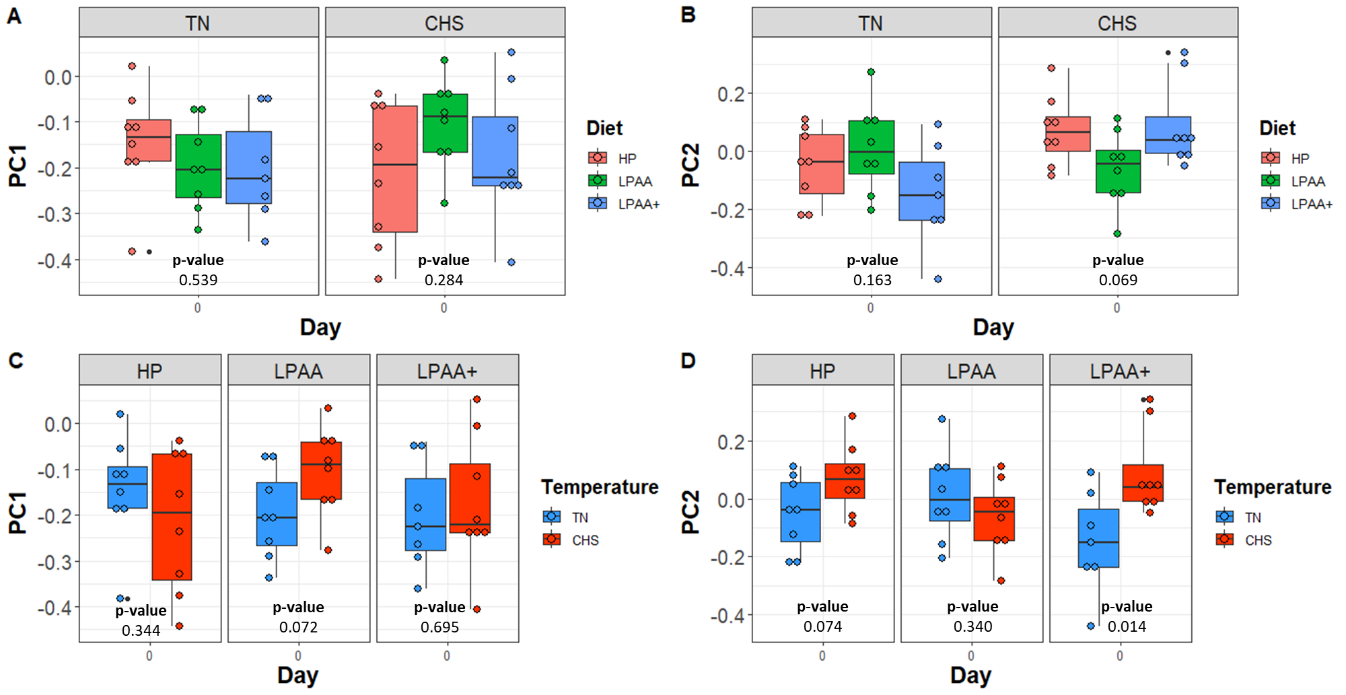

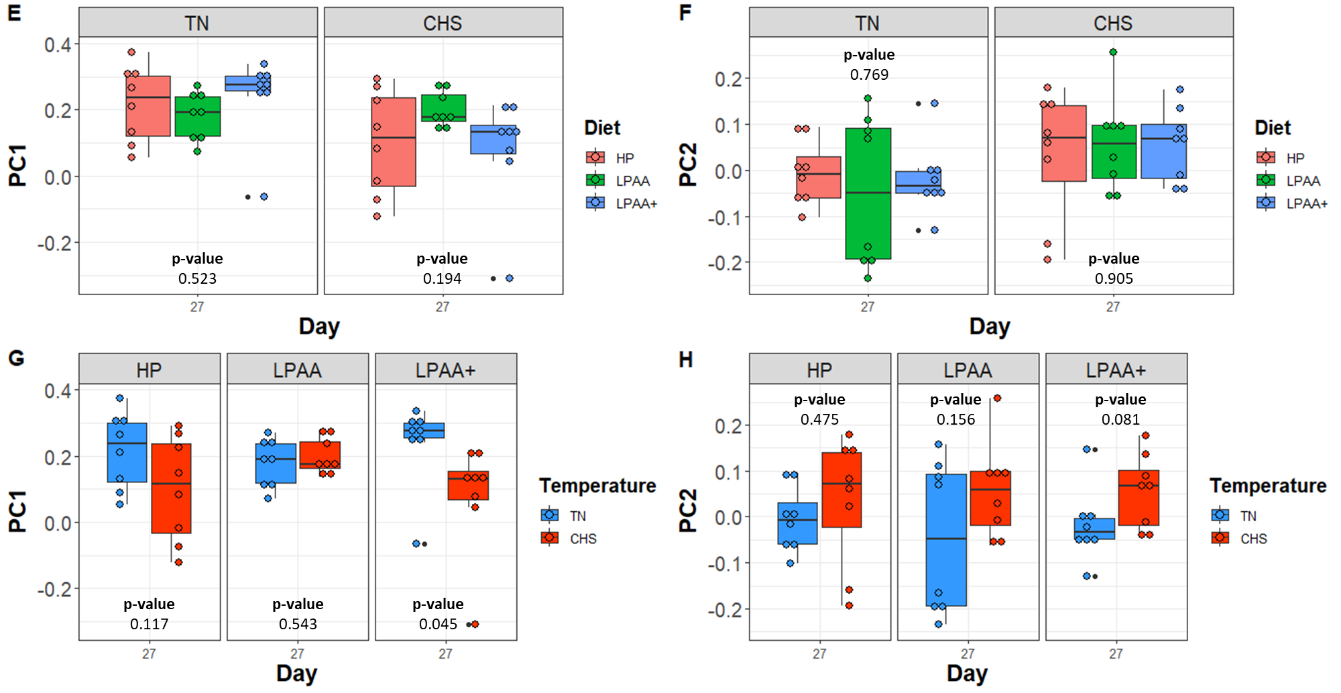
****Supplementary Figure 1.** Beta-diversity decomposition analysis for pig's fecal samples across experimental days (day 0 and day 27) and treatments. Principal coordinates (PC1 and PC2) were analyzed separately to examine the dispersion and volatility of microbial communities across treatments. When the interaction Diet × Temperature was significant based on the PERMANOVA model (p < 0.05), a one-way ANOVA analysis was used to measure the effect of Diet and Temperature, for each PC on each day. (**A**) e (**C**) PC1 on day 0, (**B**) e (**D**) PC2 on day 0, (**E**) e (**G**) PC1 on day 27, (**F**) e (**H**) PC2 on day 27. Temperatures were: thermoneutrality (TN, 24 h ~ 21.9 °C), and cyclic heat stress (CHS, 12 h ~ 33.8 °C and 12 h ~ 22.4 °C). Diets were HP = high crude protein (CP) diet; LPAA = low CP-free amino acid (AA) supplemented diet; LPAA+ = low CP-free AA-supplemented diets and digestible Lys level (+20%), and Lys:AA ratios above recommendations.


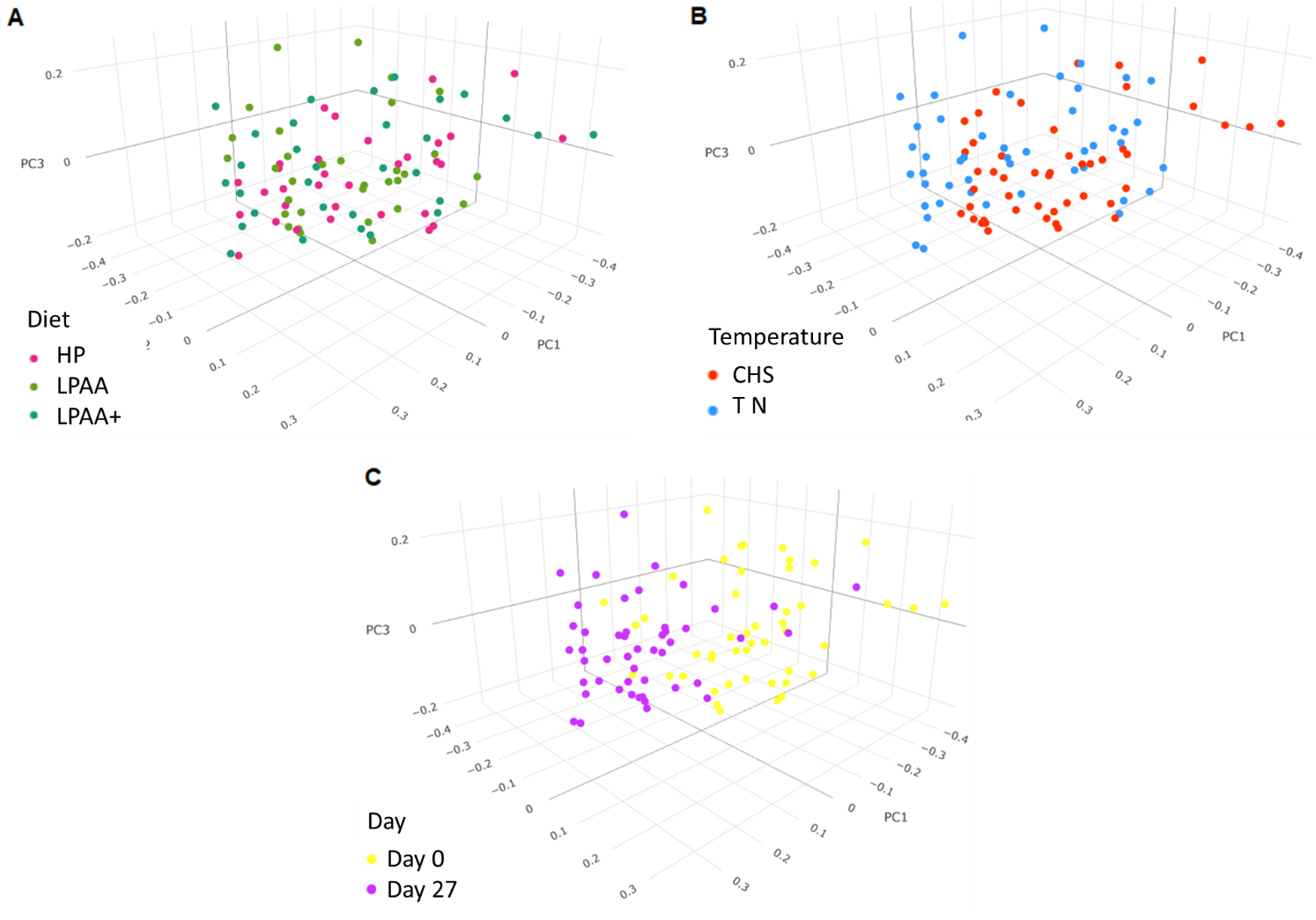


**Supplementary Figure 2.** A 3D plot of the three major axes generated by principal coordinate analysis (PC) of beta diversity analysis of the pig’s fecal microbiome by considering all samples. In the scatter plot, the color indicates groupings according to Diet (**A**), Temperature (**B**), and Day (**C**). Housing temperatures were: thermoneutrality (TN, 24 h ~ 21.9 °C), and cyclic heat stress (CHS, 12 h ~ 33.8 °C and 12 h ~ 22.4 °C). Diets were HP = high crude protein (CP) diet; LPAA = low CP-free amino acid (AA) supplemented diet; LPAA+ = low CP-free AA-supplemented diets and digestible Lys level (+20%), and Lys:AA ratios above recommendations. Days were 0 (beginning of the experiment) and 27 (end of the experiment). The sample size used to perform the 3D plotting was: HP (*n*=16), LPAA (*n*=16), LPAA+ (*n*=15), TN (*n*=47), CHS (*n*=48), Day 0 (*n*=47), Day 27 (*n*=48).


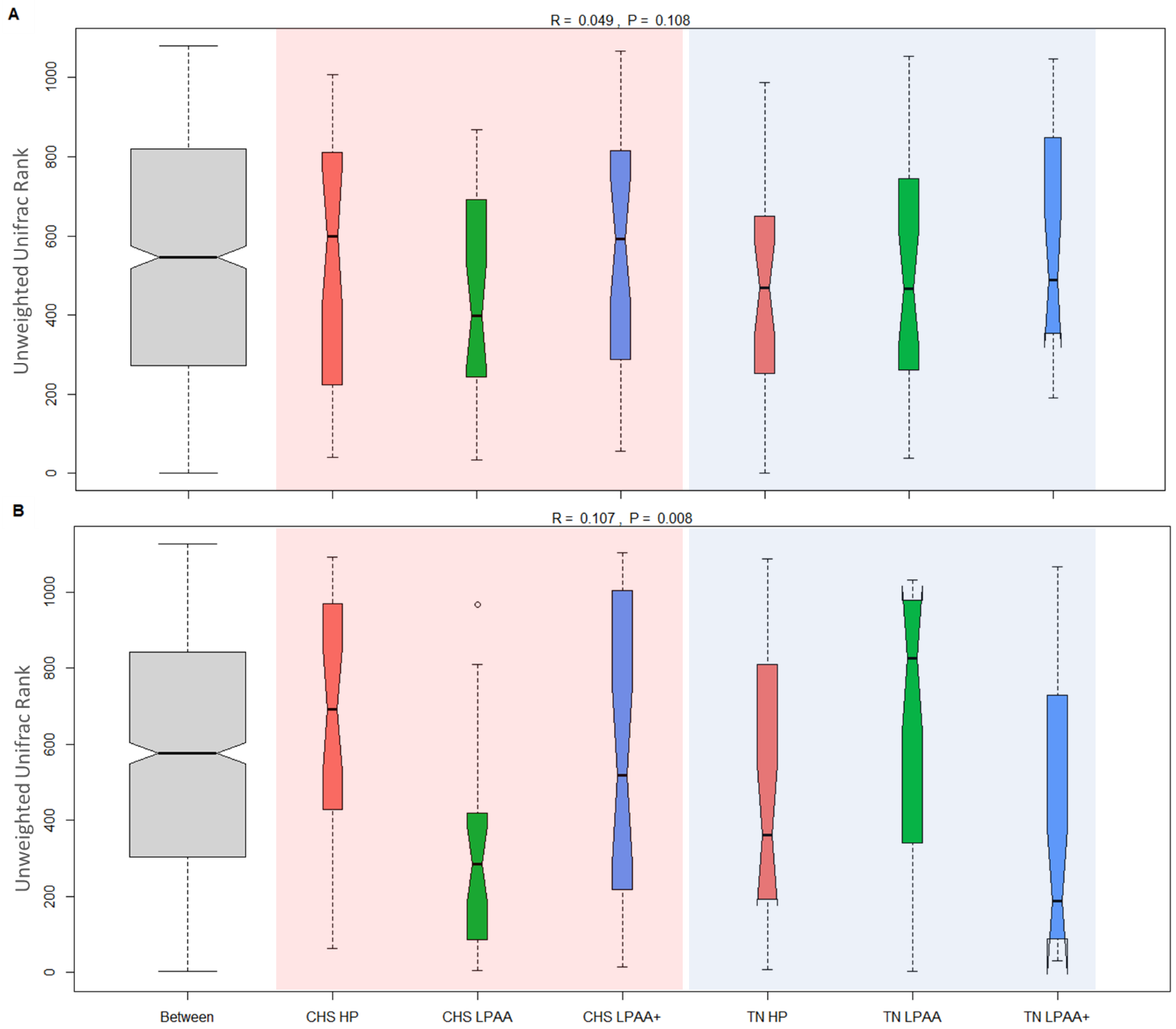


**Supplementary Figure 3.** Pig’s fecal content analysis of similarity (ANOSIM) on Day 0 (**A**) and Day 27 (**B**). The ANOSIM results were modeled based on the Bray-Curtis distance (beta-diversity) among the experimental treatments (groups) as explanatory variables. A dissimilarity matrix was generated with the vegdist() in R from the Vegan package. In brief, R > 0 indicates a difference of inter-treatment groups, whereas R < 0 indicates that the difference of intra-treatment groups was greater than that of inter-treatment groups. P indicates the reliability of the statistical analysis in which p < 0.05 represents a significant difference. When treatment groups are significantly different (p < 0.05) in their microbial community composition, then compositional dissimilarities between the treatment groups are greater than those within the treatment groups (as described here https://www.rdocumentation.org/packages/vegan/versions/2.3-5/topics/anosim). On the y-axis, Unweighted UniFrac is the rank of dissimilarity entry. On the x-axis are all individual fecal samples and the between-groups (treatments) entry. The higher the ANOSIM statistic R (value closer to 1), the more dissimilar the communities between treatments; and vice-versa (see examples here https://jkzorz.github.io/2019/06/11/ANOSIM-test.html; <https://sites.google.com/site/mb3gustame/hypothesis-tests/anosim>).


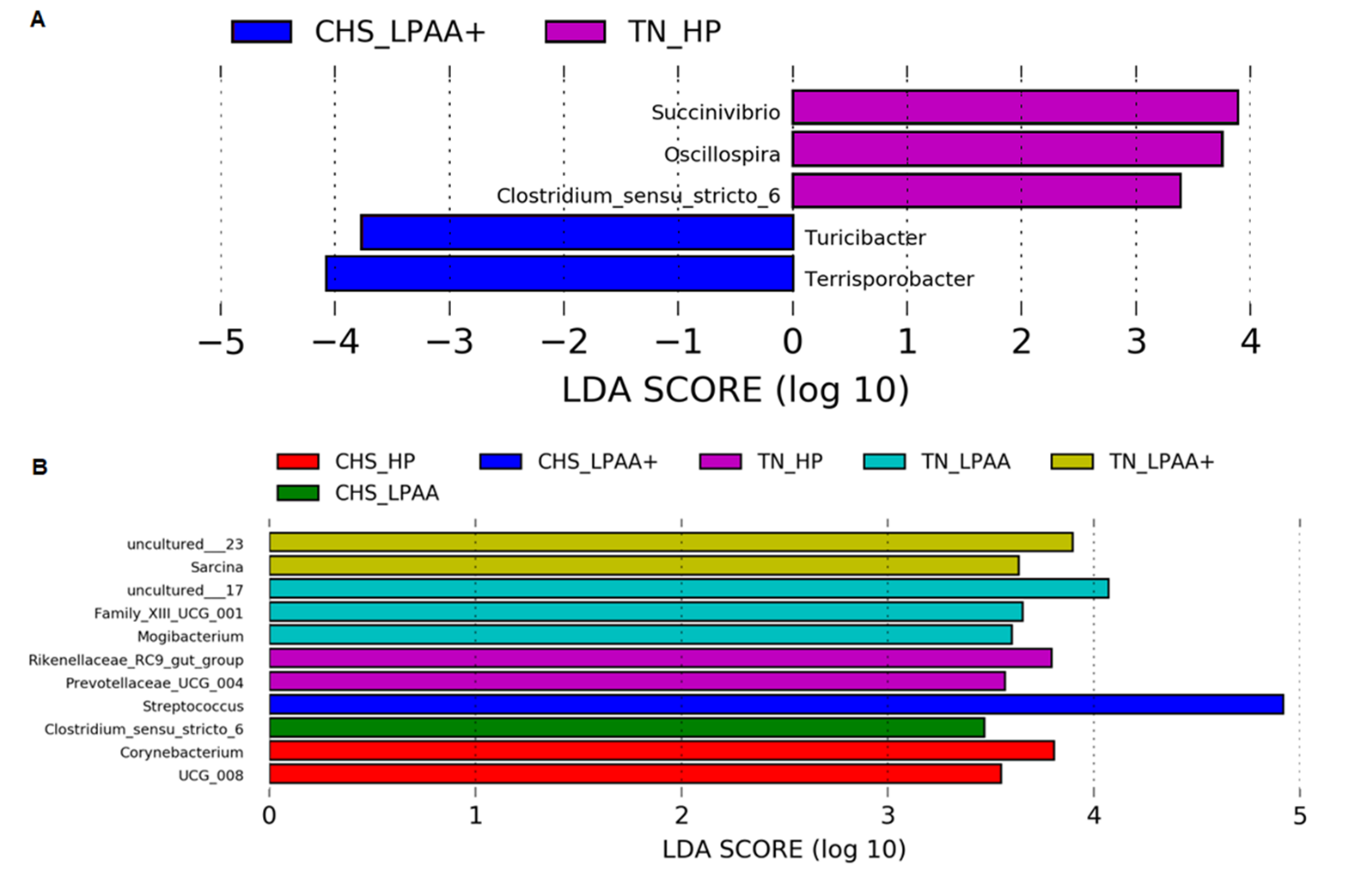
**Supplementary Figure 4.** Linear Discriminant Analysis (LDA) Effect Size (LeFse). Histogram of the LDA scores illustrating the differentially abundant taxonomic biomarkers [Δchange transformed, log_2_ (f + 1)] of pigs according to treatment. The Δchange was calculated as follows: Δchange (%) = (relative abundance on day 27 - relative abundance on day 0) / relative abundance on day 0 for all samples according to the treatment. The cut-off for LDA was set at 2. Treatments were built according to a 2 × 3 factorial arrangement with two housing temperatures (TN and CHS) and three diets (HP, LPAA, LPAA+). Temperatures were: thermoneutrality (TN, 24 h ~ 21.9 °C), and cyclic heat stress (CHS, 12 h ~ 33.8 °C and 12 h ~ 22.4 °C). Diets were: HP = high crude protein (CP) diet; LPAA = low CP-free amino acid (AA) supplemented diet; LPAA+ = low CP-free AA-supplemented diets and digestible Lys level (+20%), and Lys:AA ratios above recommendations. The LDA score represents the extent to which taxonomic biomarkers differ between treatments on day 0 (**A**) and on day 27 (**B**).


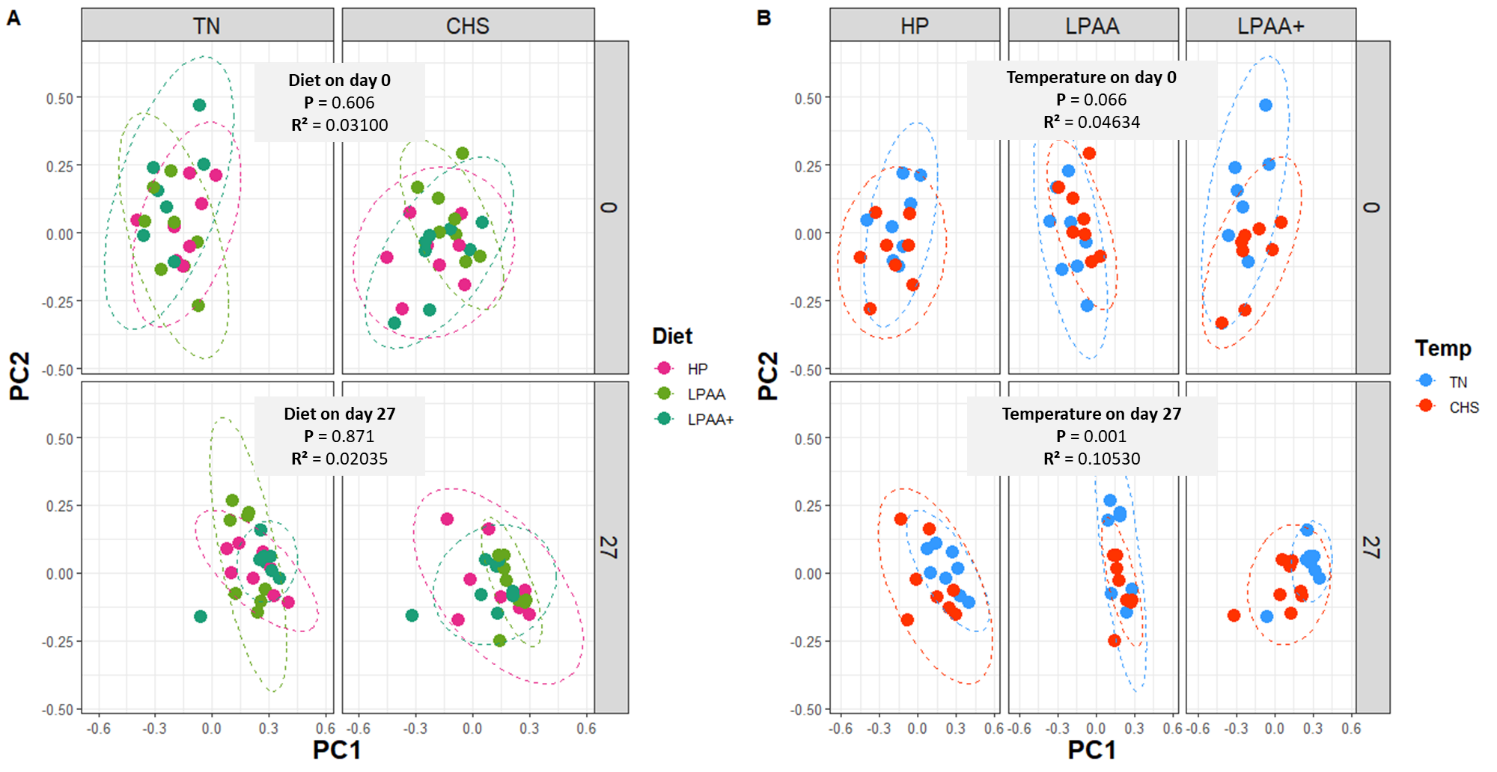


**Supplementary Figure 5.** Beta-diversity analysis of the fecal microbiome composition after removing noising taxa. Were assumed as noising taxa those that were not significant based on taxonomic biomarker relative abundance of the most abundant taxa (cut-off > 2%), structure of core community (Figure 5), and Linear Discriminant Analysis, Effect Size (Supplementary Figure 4). The Bray-Curtis distance matrix was used to calculate the beta diversity between treatments. Housing temperatures were: thermoneutrality (TN, 24 h ~ 21.9 °C), and cyclic heat stress (CHS, 12 h ~ 33.8 °C and 12 h ~ 22.4 °C). Diets were: HP = high crude protein (CP) diet; LPAA = low CP-free amino acid (AA) supplemented diet; LPAA+ = low CP-free AA-supplemented diets and digestible Lys level (+20%), and Lys:AA ratios above recommendations. Two principal coordinates (PC1, PC2) are shown for fecal samples at the beginning (day 0) and at the end (day 27) of the experiment, for each housing temperature (**A**), and diet (**B**). Testing all factors (and interactions) by PERMANOVA model supports Diet × Temperature interactions (p = 0.018, R² = 0.0321), Temperature (p = 0.005, R² = 0.0334), and Day (p = 0.001, R² = 0.3395) effects, but not Diet (p = 0.534, R² = 0.0108) effects (Shown in Supplementary Table 6). Testing factors by PERMANOVA model on day 0 (Shown in Supplementary Table 7) supports Diet × Temperature interactions (p = 0.016, R² = 0.10218), but not the single effect of Diet (p = 0.606, R² = 0.03100) or Temperature (p = 0.066, R² = 0.04634). Testing factors by PERMANOVA model on day 27 (Shown in Supplementary Table 8) support the Temperature effect (p = 0.001, R² = 0.10530) but not the Diet effect (p = 0.871, R² = 0.02035) or their interactions (p = 0.432, R² = 0.03927).

**Supplementary Table 1.** Alpha-diversity analysis (Shannon’s index) of the fecal microbiome composition of pigs. Statistical analysis was done using the one-way ANOVA to measure the effect of each studied factor (Temperature, Diet, and Day) and their interactions.

| **Factors** | **Df** | **Sum Sq** | **Means Sq** | **F value** | **Pr(>F)** |
| --- | --- | --- | --- | --- | --- |
| Day | 1 | 1.38 | 1.3801 | 17.271 | < 0.001 |
| Diet | 2 | 0.142 | 0.0709 | 0.887 | 0.416 |
| Temperature | 1 | 0.074 | 0.0738 | 0.923 | 0.340 |
| Day × Diet | 2 | 0.097 | 0.0487 | 0.609 | 0.546 |
| Day × Temperature | 1 | 0.069 | 0.0686 | 0.858 | 0.357 |
| Diet × Temperature | 2 | 0.613 | 0.3066 | 3.837 | 0.026 |
| Day × Diet × Temperature | 2 | 0.24 | 0.1201 | 1.503 | 0.228 |
| Residuals | 83 | 6.633 | 0.0799 |  |  |

**Supplementary Table 2.** Alpha-diversity analysis (Simpson’s D index) of the fecal microbiome composition of pigs. Statistical analysis was done using the one-way ANOVA to measure the effect of each studied factor (Temperature, Diet, and Day) and their interactions.

| **Factors** | **Df** | **Sum Sq** | **Means Sq** | **F value** | **Pr(>F)** |
| --- | --- | --- | --- | --- | --- |
| Day | 1 | 0.0515 | 0.05154 | 10.819 | 0.002 |
| Diet | 2 | 0.0017 | 0.00085 | 0.179 | 0.836 |
| Temperature | 1 | 0.0017 | 0.00167 | 0.35 | 0.556 |
| Day × Diet | 2 | 0.0085 | 0.00424 | 0.889 | 0.415 |
| Day × Temperature | 1 | 0.0003 | 0.00028 | 0.059 | 0.809 |
| Diet × Temperature | 2 | 0.0381 | 0.01903 | 3.996 | 0.022 |
| Day × Diet × Temperature | 2 | 0.0159 | 0.00796 | 1.671 | 0.194 |
| Residuals | 83 | 0.3954 | 0.00476 |  |  |

**Supplementary Table 3.** Permutational multivariate analysis of variance (PERMANOVA) to test the effect of Day, Diet, Temperature, and their interactions on pig’s fecal microbiota.

| Factors | Df | Sum Sq | R2 | F value | Pr(>F) |
| --- | --- | --- | --- | --- | --- |
| Temperature | 1 | 0.2818 | 0.03002 | 4.2806 | 0.006 |
| Diet | 2 | 0.1193 | 0.0127 | 0.9057 | 0.487 |
| Day | 1 | 2.8772 | 0.30642 | 43.6977 | 0.001 |
| Temperature × Diet | 2 | 0.2977 | 0.03171 | 2.2609 | 0.028 |
| Temperature × Day | 1 | 0.1302 | 0.01386 | 1.9771 | 0.090 |
| Diet × Day | 2 | 0.068 | 0.00724 | 0.5165 | 0.840 |
| Temperature × Diet × Day | 2 | 0.1504 | 0.01602 | 1.1425 | 0.320 |
| Residual | 83 | 5.4649 | 0.58202 |  |  |
| Total | 94 | 9.3896 | 1 |  |  |

**Supplementary Table 4.** Permutational multivariate analysis of variance (PERMANOVA) to test the effect of Diet, Temperature, and their interactions on pig’s fecal microbiota at the beginning of the experiment (Day 0).

| Factors | Df | Sum Sq | R2 | F value | Pr(>F) |
| --- | --- | --- | --- | --- | --- |
| Temperature | 1 | 0.1462 | 0.04112 | 2.0166 | 0.081 |
| Diet | 2 | 0.1021 | 0.02873 | 0.7044 | 0.703 |
| Temperature × Diet | 2 | 0.3344 | 0.09406 | 2.3062 | 0.017 |
| Residual | 41 | 2.9722 | 0.83609 |  |  |
| Total | 46 | 3.5549 | 1 |  |  |

**Supplementary Table 5.** Permutational multivariate analysis of variance (PERMANOVA) to test the effect of Diet, Temperature, and their interactions on pig’s fecal microbiota at the end of the experiment (Day 27).

| Factors | Df | Sum Sq | R2 | F value | Pr(>F) |
| --- | --- | --- | --- | --- | --- |
| Temperature | 1 | 0.25932 | 0.08777 | 4.3694 | 0.004 |
| Diet | 2 | 0.08887 | 0.03008 | 0.7487 | 0.696 |
| Temperature × Diet | 2 | 0.11368 | 0.03847 | 0.9577 | 0.467 |
| Residual | 42 | 2.49271 | 0.84368 |  |  |
| Total | 47 | 2.95458 | 1 |  |  |

**Supplementary Table 6.** Permutational multivariate analysis of variance (PERMANOVA) to test the effect of Day, Diet, Temperature, and their interactions on pig’s fecal microbiota after removing noising taxa¹.

| Factors | Df | Sum Sq | R2 | F value | Pr(>F) |
| --- | --- | --- | --- | --- | --- |
| Temperature | 1 | 0.2982 | 0.0334 | 5.0743 | 0.005 |
| Diet | 2 | 0.096 | 0.0108 | 0.8169 | 0.534 |
| Day | 1 | 3.0319 | 0.3395 | 51.601 | 0.001 |
| Temperature × Diet | 2 | 0.2862 | 0.0321 | 2.4356 | 0.018 |
| Temperature × Day | 1 | 0.1406 | 0.0158 | 2.3937 | 0.074 |
| Diet × Day | 2 | 0.0536 | 0.0060 | 0.4561 | 0.868 |
| Temperature × Diet × Day | 2 | 0.1481 | 0.0166 | 1.2602 | 0.256 |
| Residual | 83 | 4.8768 | 0.546 |  |  |
| Total | 94 | 8.9315 | 1 |  |  |

¹ Were assumed as noising taxa those that were not significant based on taxonomic biomarker relative abundance of the most abundant taxa (cut-off > 2%), structure of core community (Figure 5), and Linear Discriminant Analysis, Effect Size (Supplementary Figure 4).

**Supplementary Table 7.** Permutational multivariate analysis of variance (PERMANOVA) to test the effect of Diet, Temperature, and their interactions on pig’s fecal microbiota at the beginning of the experiment (Day 0) after removing noising taxa¹.

| Factors | Df | Sum Sq | R2 | F value | Pr(>F) |
| --- | --- | --- | --- | --- | --- |
| Temperature | 1 | 0.1490 | 0.04634 | 2.3156 | 0.066 |
| Diet | 2 | 0.0997 | 0.03100 | 0.7746 | 0.606 |
| Temperature × Diet | 2 | 0.3286 | 0.10218 | 2.5530 | 0.016 |
| Residual | 41 | 2.6386 | 0.82048 |  |  |
| Total | 46 | 3.2159 | 1.00000 |  |  |

¹ Were assumed as noising taxa those that were not significant based on taxonomic biomarker relative abundance of the most abundant taxa (cut-off > 2%), structure of core community (Figure 5), and Linear Discriminant Analysis, Effect Size (Supplementary Figure 4).

**Supplementary Table 8.** Permutational multivariate analysis of variance (PERMANOVA) to test the effect of Diet, Temperature, and their interactions on pig’s fecal microbiota at the end of the experiment (Day 27) after removing noising taxa¹.

| Factors | Df | Sum Sq | R2 | F value | Pr(>F) |
| --- | --- | --- | --- | --- | --- |
| Temperature | 1 | 0.2822 | 0.10530 | 5.2961 | 0.001 |
| Diet | 2 | 0.0545 | 0.02035 | 0.5116 | 0.871 |
| Temperature × Diet | 2 | 0.1053 | 0.03927 | 0.9876 | 0.432 |
| Residual | 42 | 2.2382 | 0.83508 |  |  |
| Total | 47 | 2.6803 | 1.00000 |  |  |

¹ Were assumed as noising taxa those that were not significant based on taxonomic biomarker relative abundance of the most abundant taxa (cut-off > 2%), structure of core community (Figure 5), and Linear Discriminant Analysis, Effect Size (Supplementary Figure 4).

**Supplementary Table 9.** One-way ANOVA to test the effect of each Diet, Temperature, and their interactions on the taxa Δchange ¹.

| **Taxa** | **Temperature** | **Diet** | **Temperature × Diet** |
| --- | --- | --- | --- |
| *Acidaminococcus* | 0.134 | 0.229 | 0.115 |
| *Blautia* | 0.514 | 0.500 | 0.996 |
| *Catenibacterium* | 0.931 | 0.799 | 0.191 |
| *Christensenellaceae R-7* | 0.343 | 0.449 | 0.190 |
| *Clostridium sensu stricto 1* | 0.594 | 0.429 | 0.412 |
| *Clostridium sensu stricto 6* | 0.995 | 0.797 | 0.104 |
| *Corynebacterium* | 0.001 | 0.587 | 0.861 |
| *Dorea* | 0.804 | 0.373 | 0.664 |
| *Eubacterium hallii* | 0.045 | 0.804 | 0.720 |
| *Eubacterium nodatum* | 0.982 | 0.732 | 0.328 |
| *Intestinibacter* | 0.447 | 0.582 | 0.728 |
| *Lachnospiraceae NK4A136* | 0.463 | 0.355 | 0.594 |
| *Lactobacillus* | 0.560 | 0.587 | 0.053 |
| *Megasphaera* | 0.567 | 0.280 | 0.387 |
| *Mogibacterium* | 0.401 | 0.816 | 0.008 |
| *Oscillospira* | 0.728 | 0.025 | 0.804 |
| *Peptococcus* | 0.728 | 0.459 | 0.455 |
| *Phascolarctobacterium* | 0.161 | 0.838 | 0.598 |
| *Prevotella* | 0.029 | 0.664 | 0.220 |
| *Prevotellaceae NK3B31* | 0.976 | 0.285 | 0.543 |
| *Prevotellaceae UCG 004* | 0.093 | 0.196 | 0.482 |
| *Prevotellaceae UCG 001* | 0.307 | 0.678 | 0.818 |
| *Rikenellaceae RC9* | 0.328 | 0.244 | 0.479 |
| *Ruminococcus* | 0.486 | 0.499 | 0.299 |
| *Ruminococcus gauvreauii* | 0.901 | 0.653 | 0.544 |
| *Sarcina* | 0.230 | 0.818 | 0.212 |
| *Solobacterium* | 0.012 | 0.106 | 0.200 |
| *Streptococcus* | 0.001 | 0.594 | 0.407 |
| *Subdoligranulum* | 0.423 | 0.326 | 0.776 |
| *Succinivibrio* | 0.082 | 0.375 | 0.092 |
| *Syntrophococcus* | 0.108 | 0.842 | 0.676 |
| *Terrisporobacter* | 0.285 | 0.402 | 0.430 |
| *Treponema* | 0.568 | 0.793 | 0.143 |
| *Turicibacter* | 0.040 | 0.494 | 0.258 |

¹ Analysis was done based on Δchange of transformed [log_2_ (f + 1)] taxonomic biomarkers of the most abundant taxa (cut-off > 2%), structure of core community (Figure 5), and Linear Discriminant Analysis, Effect Size (Supplementary Figure 4). The Δchange was calculated as follows: Δchange (%) = (relative abundance on day 27 - relative abundance on day 0) / relative abundance on day 0.
